# A 4-hydroxybenzoate 3-hydroxylase mutant enables 4-amino-3-hydroxybenzoic acid production from glucose in *Corynebacterium glutamicum*

**DOI:** 10.1186/s12934-023-02179-y

**Published:** 2023-08-29

**Authors:** Kyoshiro Nonaka, Tatsuya Osamura, Fumikazu Takahashi

**Affiliations:** Biological Science Research, Kao Corporation, 1334 Minato, Wakayama, Wakayama 640-8580 Japan

**Keywords:** 4-Amino-3-hydroxybenzoic acid, 4-Aminobenzoic acid, Polybenzoxazole, Artificial biosynthetic pathway, Shikimate pathway, *Corynebacterium glutamicum*, Flavoprotein monooxygenases, 4-Hydroxybenzoate 3-hydroxylase, Enzyme engineering, Genome-scale metabolic modeling

## Abstract

**Background:**

Microbial production of aromatic chemicals is an attractive method for obtaining high-performance materials from biomass resources. A non-proteinogenic amino acid, 4-amino-3-hydroxybenzoic acid (4,3-AHBA), is expected to be a precursor of highly functional polybenzoxazole polymers; however, methods for its microbial production have not been reported. In this study, we attempted to produce 4,3-AHBA from glucose by introducing 3-hydroxylation of 4-aminobenzoic acid (4-ABA) into the metabolic pathway of an industrially relevant bacterium, *Corynebacterium glutamicum.*

**Results:**

Six different 4-hydroxybenzoate 3-hydroxylases (PHBHs) were heterologously expressed in *C. glutamicum* strains, which were then screened for the production of 4,3-AHBA by culturing with glucose as a carbon source. The highest concentration of 4,3-AHBA was detected in the strain expressing PHBH from *Caulobacter vibrioides* (*Cv*PHBH). A combination of site-directed mutagenesis in the active site and random mutagenesis via laccase-mediated colorimetric assay allowed us to obtain *Cv*PHBH mutants that enhanced 4,3-AHBA productivity under deep-well plate culture conditions. The recombinant *C. glutamicum* strain expressing *Cv*PHBH^M106A/T294S^ and having an enhanced 4-ABA biosynthetic pathway produced 13.5 g/L (88 mM) 4,3-AHBA and 0.059 g/L (0.43 mM) precursor 4-ABA in fed-batch culture using a nutrient-rich medium. The culture of this strain in the chemically defined CGXII medium yielded 9.8 C-mol% of 4,3-AHBA from glucose, corresponding to 12.8% of the theoretical maximum yield (76.8 C-mol%) calculated using a genome-scale metabolic model of *C. glutamicum*.

**Conclusions:**

Identification of PHBH mutants that could efficiently catalyze the 3-hydroxylation of 4-ABA in *C. glutamicum* allowed us to construct an artificial biosynthetic pathway capable of producing 4,3-AHBA on a gram-scale using glucose as the carbon source. These findings will contribute to a better understanding of enzyme-catalyzed regioselective hydroxylation of aromatic chemicals and to the diversification of biomass-derived precursors for high-performance materials.

**Supplementary Information:**

The online version contains supplementary material available at 10.1186/s12934-023-02179-y.

## Background

Microbial production of aromatic chemicals from biomass-derived carbon sources is an attractive method for obtaining renewable precursors for high-performance materials in the face of climate change and depletion of fossil fuel resources [1–3]. A non-proteinogenic amino acid, 4-amino-3-hydroxybenzoic acid (4,3-AHBA), retains *ortho*-aminophenol and carboxylic acid groups in its molecular structure; therefore, it is expected to be a precursor for polybenzoxazole (PBO), a class of highly functional materials with excellent thermal stability and mechanical strength formed by a ring-closing condensation reaction of these two functional groups [4, 5]. Recent studies demonstrated that 3-amino-4-hydroxybenzoic acid (3,4-AHBA), a structural isomer of 4,3-AHBA, can be biosynthesized from biomass-derived carbon sources using recombinant *Corynebacterium glutamicum*, possessing two-step enzymatic reactions originally identified in *Streptomyces griseus* [6–8], and can be used as a precursor for poly(2,5-benzoxazole-co-2,5-benzimidazole) plastic films [9]. In contrast, to the best of our knowledge, the microbial production of 4,3-AHBA from biomass-derived carbon sources has not been reported.

In the chemical production process, stoichiometric reduction of various nitroaromatic compounds has traditionally been used for the industrial production of corresponding amines [10–12]. High-yield (typically >99%) catalytic reduction using noble metal elements has also been investigated to address the concerns regarding the environmental burden of the large amount of waste generated in the stoichiometric reduction process [11–13]. However, 3-hydroxy-4-nitrobenzoic acid, a reactant corresponding to 4,3-AHBA, is not industrially produced from renewable sources at low costs due to the lack of commercial production as a bulk chemical.

So far, there have been several reports on the biocatalytic formation of 4,3-AHBA, such as microbial conversion of 4-hydroxylaminobenzoic acid [14] and enzymatic 3-hydroxylation of 4-aminobenzoic acid (4-ABA) in vitro [15, 16]. In particular, biochemical studies on 4-hydroxybenzoate 3-hydroxylases (PHBHs, EC 1.14.13.2) from *Pseudomonas aeruginosa* (*Pa*PHBH) and *Pseudomonas fluorescens* (*Pf*PHBH) using 4-ABA as a structural analog of 4-hydroxybenzoic acid (4-HBA) have shown that these enzymes exhibit low 3-hydroxylation activity toward 4-ABA [15, 16]. In nature, various microorganisms biosynthesize 4-ABA as a precursor of folate via 4-amino-4-deoxychorismate (ADC) by the action of ADC synthase (EC 2.6.1.85) and ADC lyase (EC 4.1.3.38) on chorismic acid, the final product of the shikimate pathway [17]. The shikimate pathway constitutes the upstream reactions of the biosynthetic pathways of aromatic amino acids, such as *L*-phenylalanine, *L*-tyrosine, and *L*-tryptophan, and is preferably used to produce various aromatic chemicals from biomass-derived carbon sources [1, 2]. Indeed, efficient production of 4-ABA (43 g/L) from glucose has been achieved using genetically engineered *C. glutamicum* [17]. In this context, we hypothesized that 4,3-AHBA could be obtained from glucose as a biomass-derived carbon source by developing a PHBH that efficiently catalyzes the 3-hydroxylation of 4-ABA and expressing it in a suitable host microorganism.

*C. glutamicum* was deemed as a suitable host microorganism because it is relatively resistant to several aromatic chemicals, such as 4-ABA (up to 400 mM) [17], 4-HBA (up to 300 mM) [18], and protocatechuic acid (PCA, up to 500 mM) [19]. The high tolerance to aromatic chemicals is considered advantageous for their production [20]. *C. glutamicum* has been used to produce various benzoic acids and their derivatives, including 4-ABA [17], 4-HBA [18, 21, 22], PCA [19, 22, 23], 2-hydroxybenzoic acid [22], 3-hydroxybenzoic acid [22], 3,4-AHBA [7, 8], anthranilic acid [24], methyl anthranilic acid [25], *N*-methyl anthranilic acid [26], 4-hydroxybenzaldehyde [27], and vanillin [27]. In addition, *C. glutamicum* has been widely used for the industrial production of amino acids, such as *L*-glutamic acid and *L*-lysine; the repertoire of products has been extended to include commodity chemicals and cosmetics through metabolic engineering techniques [28–31]. Furthermore, *C. glutamicum* does not contain endotoxins and secretes only a few endogenous proteins into its extracellular culture medium [32]. These properties are advantageous as they simplify the purification process for the production of both proteins and chemicals.

**Fig. 1:**
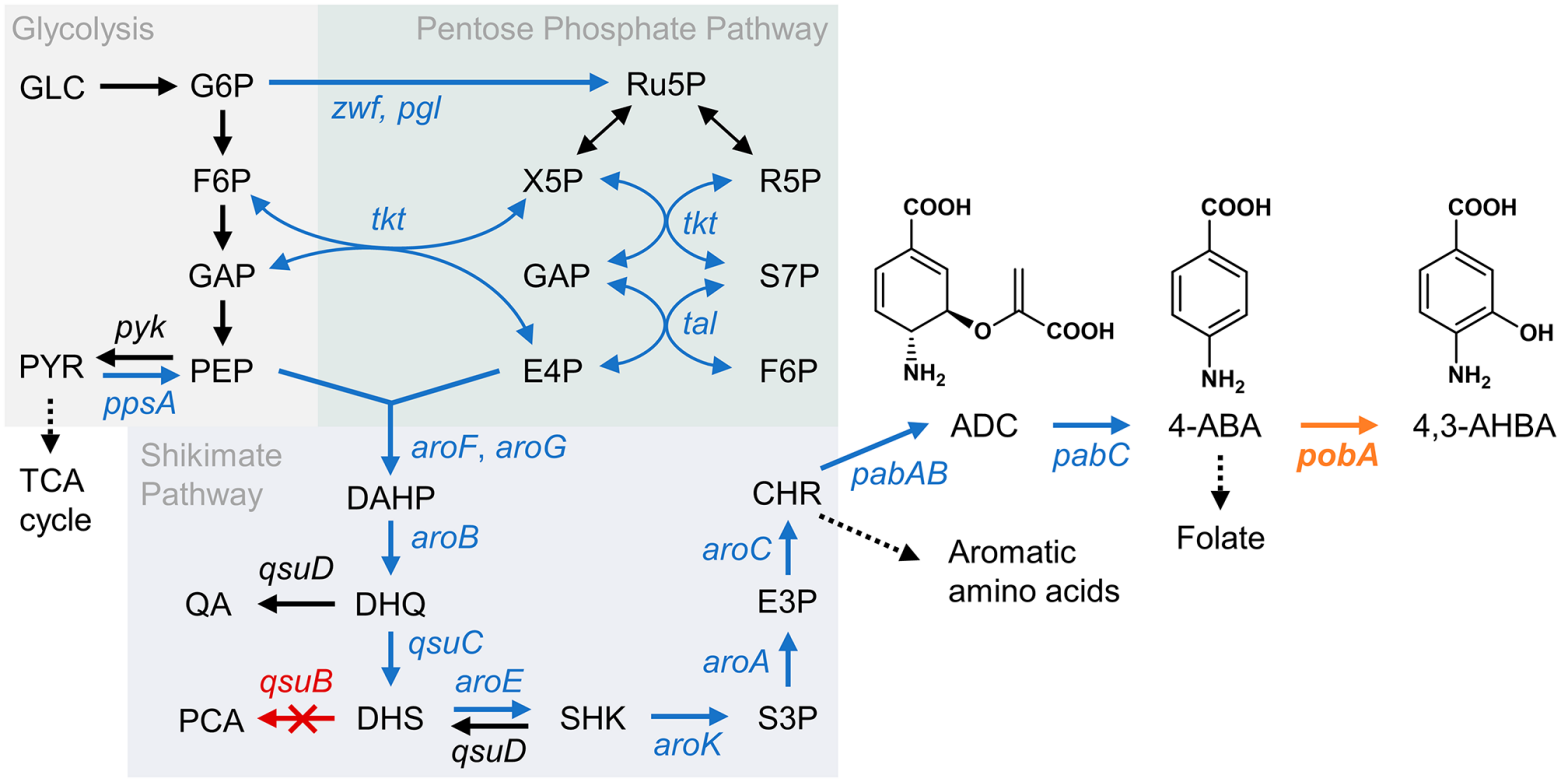
Schematic representation of the artificial pathway for the production of 4,3-AHBA from glucose in *Corynebacterium glutamicum*. Italic text indicates relevant genes in the pathway. Blue text indicates genes whose corresponding protein expression was enhanced by the constitutive P_*tuf*_ promoter. Orange text indicates the heterologous *pobA* gene encoding 4-hydroxybenzoate 3-hydroxylase (PHBH), which is expressed for the 3-hydroxylation of 4-ABA. Red text indicates the disrupted gene. The indicated genes encode the following enzymes: *zwf*, glucose 6-phosphate dehydrogenase; *pgl*, 6-phosphogluconolactonase; *tkt*, transketolase; *tal*, transaldolase; *pyk*, pyruvate kinase; *ppsA*, phosphoenolpyruvate synthase; *aroF* and *aroG*, DAHP synthase; *aroB*, 3-dehydroquinate synthase; *qsuC*, 3-dehydroquinate dehydratase; *qsuB*, dehydroshikimate dehydratase; *qsuD* and *aroE*, shikimate dehydrogenase; *aroK*, shikimate kinase; *aroA*, 5-enolpyruvylshikimate-3-phosphate synthase; *aroC*, chorismate synthase; *pabAB*, ADC synthase; *pabC*, ADC lyase; and *pobA*, PHBH. The abbreviations of the metabolites are: GLC, glucose; G6P, glucose-6-phosphate; F6P, fructose-6-phosphate; GAP, glyceraldehyde 3-phosphate; PEP, phosphoenolpyruvate; PYR, pyruvate; TCA, tricarboxylic acid; Ru5P, ribulose-5-phosphate; X5P, xylulose-5-phosphate; R5P, ribose-5-phosphate; S7P, sedoheptulose-7-phosphate; E4P, erythrose-4-phosphate; DAHP, 3-deoxy-D-arabino-heptulosonate-7-phosphate; DHQ, 3-dehydroquinate; QA, quinate; DHS, 3-dehydroshikimate; PCA, protocatechuate; SHK, shikimate; S3P, shikimate-3-phosphate; E3P, 5-enolpyruvylshikimate-3-phosphate; CHR, chorismate; ADC, 4-amino-4-deoxychorismate; 4-ABA, 4-aminobenzoic acid; and 4,3-AHBA, 4-amino-3-hydroxybenzoic acid

Here, we report the production of 4,3-AHBA from glucose using *C. glutamicum* strains genetically engineered to express PHBH mutants that catalyze the 3-hydroxylation of 4-ABA (Fig. 1). After screening six different PHBHs and performing mutagenesis in a selected one, we obtained several PHBH mutants that efficiently produced 4,3-AHBA in *C. glutamicum*. Subsequently, recombinant *C. glutamicum* strains expressing the PHBH mutants with an improved 4-ABA biosynthetic pathway were constructed and tested for gram-scale production of 4,3-AHBA using a fed-batch culture method. The theoretical maximum yield of 4,3-AHBA production was calculated using a genome-scale metabolic model of *C. glutamicum*, and a fed-batch culture of the selected strain was performed in a chemically defined medium.

## Results

### Screening of wild type PHBHs for 3-hydroxylation of 4-ABA in *C. glutamicum*

To obtain a wild type enzyme suitable for the 3-hydroxylation of 4-ABA, several *C. glutamicum* strains expressing heterologous PHBHs were constructed and evaluated based on the concentration of 4,3-AHBA produced in the culture supernatant.

An *Escherichia coli*/*C. glutamicum* shuttle vector pKCG_P_*tuf*__T1 was constructed to express the protein of interest in *C. glutamicum* using commercially available DNA materials. The vector has an expression cassette comprising a strong constitutive promoter (P_*tuf*_) derived from upstream of the *tuf* gene (gene number: cg0587) encoding the elongation factor TU [33] and the artificially synthesized *rrnB* T1 terminator with the same sequence as that of pVWEx1 (GenBank ID: MF034723.1). The *pobA* genes encoding PHBHs from *Bradyrhizobium diazoefficiens* (*Bd*PHBH, NCBI accession number: WP_011089160.1), *Caulobacter vibrioides* (*Cv*PHBH, WP_010920262.1), *Rhodopseudomonas palustris* (*Rp*PHBH, WP_011157287.1), *Sinorhizobium meliloti* (*Sm*PHBH, WP_010976283.1), *Cupriavidus metallidurans* (*Cm*PHBH, WP_011519894.1), and *Rhodococcus fascians* (*Rf*PHBH, WP_027494688.1) were selected from our in-house hydroxylase library, codon-optimized for *C. glutamicum*, and inserted into the expression cassette of pKCG_P_*tuf*__T1. The amino acid sequences of the six PHBHs were less than 67% identical to each other (Additional file 1: Table S1). Endogenous *pobA* in *C. glutamicum* was excluded as a candidate in this study, based on the report that it has no activity toward 4-ABA [34]. The resulting plasmids were used to construct transformed *C. glutamicum* strains, KN001–KN007 (Table 1).

**Table 1:** *Corynebacterium glutamicum* strains used in this study

Strain	Relevant characteristics	Reference
NBRC 12168	Wild type *C. glutamicum* strain identical to ATCC 13032	^a^NBRC
NBRC 12169	Wild type *C. glutamicum* strain identical to ATCC 13058	NBRC
HT23	NBRC 12168 derivate; Δ*cglIM* (cg1996), Δ*cglIR* (cg1997), Δ*cglIIR* (cg1998)	[62]
KC265	HT23 derivate; P_*tuf*__*aroG* (cg2391)	This study
KC282	KC265 derivate; P_*tuf*__*aroE3* (cg1835)	This study
KC300	KC282 derivate; P_*tuf*__*aroB* (cg1827)	This study
KC314	KC300 derivate; P_*tuf*__*aroA* (cg0873)	This study
KC315	KC314 derivate; ^b^Δ*pobA* (cg1226), P_*tuf*__*qsuC* (cg0503)	This study
KC367	KC315 derivate; ^c^P_*tuf*__*aroC* (cg1829)	This study
KC376	KC367 derivate; ^d^P_*tuf*__*tkt* (cg1774)	This study
KC408	KC376 derivate; P_*tuf*__*ppsA* (cg0644)	This study
KC525	KC408 derivate; Δ*qsuB* (cg0502)	This study
KC551	KC525 derivate; ^e^P_*tuf*__*pabAB* (cg1134)	This study
KC594	KC551 derivate; ^f^P_*tuf*__*aroG*^*D146N*^	This study
KC617	KC594 derivate; ^g^P_*tuf*__*aroF*^*P155L*^	This study
KN001	^h^Km^R^; KC551 harboring pKCG_P_*tuf*__T1	This study
KN002	Km^R^; KC551 harboring pKCG_P_*tuf*__*Bd*PHBH_T1	This study
KN003	Km^R^; KC551 harboring pKCG_P_*tuf*__*Cv*PHBH_T1	This study
KN004	Km^R^; KC551 harboring pKCG_P_*tuf*__*Rp*PHBH_T1	This study
KN005	Km^R^; KC551 harboring pKCG_P_*tuf*__*Sm*PHBH_T1	This study
KN006	Km^R^; KC551 harboring pKCG_P_*tuf*__*Cm*PHBH_T1	This study
KN007	Km^R^; KC551 harboring pKCG_P_*tuf*__*Rf*PHBH_T1	This study
KN008	Km^R^; KC551 harboring pKCG_P_*tuf*__*Cv*PHBH^Y201F^_T1	This study
KN009	Km^R^; KC551 harboring pKCG_P_*tuf*__*Cv*PHBH^Y201S^_T1	This study
KN010	Km^R^; KC551 harboring pKCG_P_*tuf*__*Cv*PHBH^Y201T^_T1	This study
KN011	Km^R^; KC551 harboring pKCG_P_*tuf*__*Cv*PHBH^T294G^_T1	This study
KN012	Km^R^; KC551 harboring pKCG_P_*tuf*__*Cv*PHBH^T294A^_T1	This study
KN013	Km^R^; KC551 harboring pKCG_P_*tuf*__*Cv*PHBH^T294V^_T1	This study
KN014	Km^R^; KC551 harboring pKCG_P_*tuf*__*Cv*PHBH^T294L^_T1	This study
KN015	Km^R^; KC551 harboring pKCG_P_*tuf*__*Cv*PHBH^T294I^_T1	This study
KN016	Km^R^; KC551 harboring pKCG_P_*tuf*__*Cv*PHBH^T294S^_T1	This study
KN017	Km^R^; KC551 harboring pKCG_P_*tuf*__*Cv*PHBH^T294C^_T1	This study
KN018	Km^R^; KC551 harboring pKCG_P_*tuf*__*Cv*PHBH^Y201F/T294S^_T1	This study
KN019	Km^R^; KC551 harboring pKCG_P_*tuf*__*Cv*PHBH^Y161S/D357V^_T1	This study
KN020	Km^R^; KC551 harboring pKCG_P_*tuf*__*Cv*PHBH^Y161S^_T1	This study
KN021	Km^R^; KC551 harboring pKCG_P_*tuf*__*Cv*PHBH^D357V^_T1	This study
KN022	Km^R^; KC551 harboring pKCG_P_*tuf*__*Cv*PHBH^M106G^_T1	This study
KN023	Km^R^; KC551 harboring pKCG_P_*tuf*__*Cv*PHBH^M106A^_T1	This study
KN024	Km^R^; KC551 harboring pKCG_P_*tuf*__*Cv*PHBH^M106V^_T1	This study
KN025	Km^R^; KC551 harboring pKCG_P_*tuf*__*Cv*PHBH^M106L^_T1	This study
KN026	Km^R^; KC551 harboring pKCG_P_*tuf*__*Cv*PHBH^M106I^_T1	This study
KN027	Km^R^; KC551 harboring pKCG_P_*tuf*__*Cv*PHBH^M106S^_T1	This study
KN028	Km^R^; KC551 harboring pKCG_P_*tuf*__*Cv*PHBH^M106T^_T1	This study
KN029	Km^R^; KC551 harboring pKCG_P_*tuf*__*Cv*PHBH^M106C^_T1	This study
KN030	Km^R^; KC551 harboring pKCG_P_*tuf*__*Cv*PHBH^M106A/T294S^_T1	This study
KN031	Km^R^; KC551 harboring pKCG_P_*tuf*__*Cv*PHBH^M106A/Y201F/T294S^_T1	This study
KN032	Km^R^; KC617 harboring pKCG_P_*tuf*__*Cv*PHBH^Y161S/D357V^_T1	This study
KN033	Km^R^; KC617 harboring pKCG_P_*tuf*__*Cv*PHBH^M106A^_T1	This study
KN034	Km^R^; KC617 harboring pKCG_P_*tuf*__*Cv*PHBH^M106A/T294S^_T1	This study
KN035	Km^R^; KC617 harboring pKCG_P_*tuf*__*Cv*PHBH^M106A/Y201F/T294S^_T1	This study

^a^ NBRC: Biological Resource Center, National Institute of Technology and Evaluation, Japan

^b^ The endogenous *pobA* (cg1226) gene encoding PHBH was disrupted to prevent potential background 3-hydroxylation

^c^ The *aroK* (cg1828) gene is downstream and constitutes an operon

^d^ The *tal* (cg1776), *zwf* (cg1778), *opcA* (cg1779), and *pgl* (cg1780) genes are downstream and constitute an operon

^e^ The *pabC* (cg1135) gene is downstream and constitutes an operon

^f^* aroG*^*D146N*^: the codon-optimized *aroG* gene variant encoding D146N mutant of DAHP synthase from *Escherichia coli*

^g^* aroF*^*P155L*^: the *aroF* (cg1129) gene variant encoding P155L mutant of DAHP synthase

^h^ Km^R^: kanamycin resistance

**Fig. 2:**
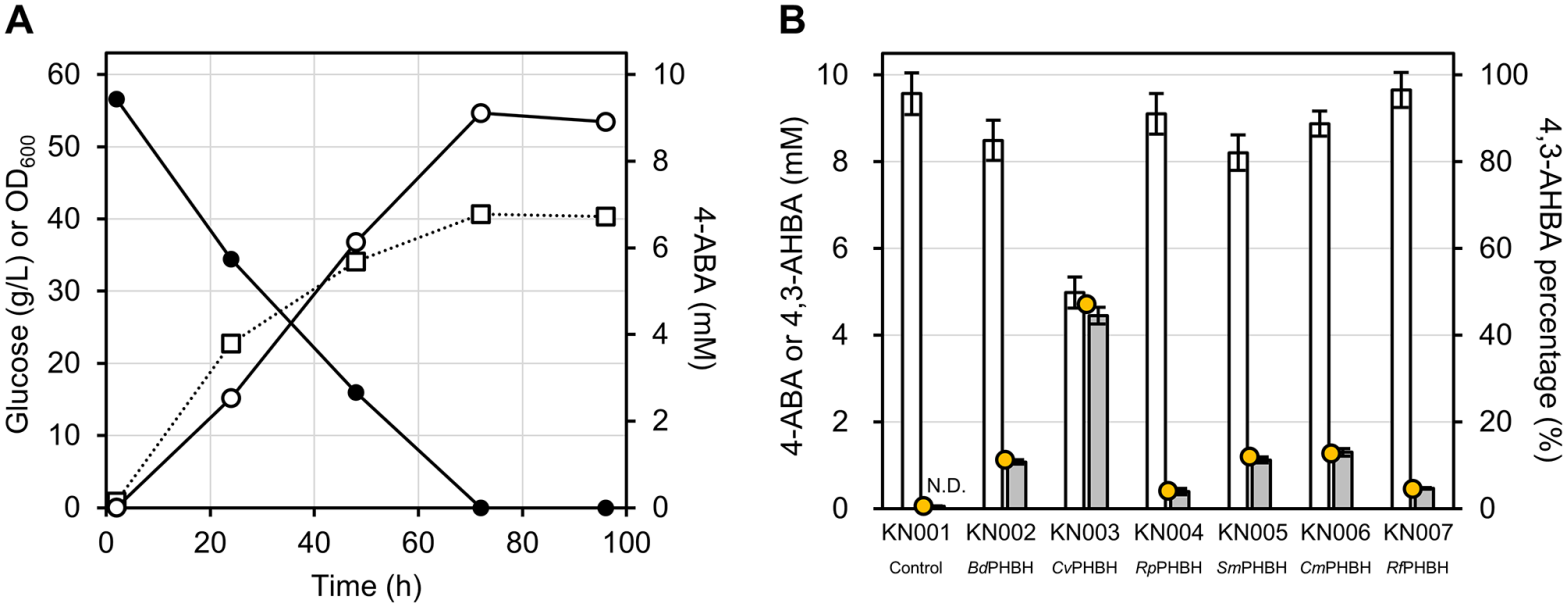
Screening of wild type 4-hydroxybenzoate 3-hydroxylases (PHBHs) under an adequate supply of 4-aminobenzoic acid (4-ABA) in *Corynebacterium glutamicum*. **A** Time-dependent 4-ABA production of KC551 in culture with nutrient-rich CGTG15 medium. Open and filled circles indicate the concentration of 4-ABA and glucose, respectively. Open squares indicate OD_600_. **B** The results of HPLC analysis of the culture supernatants of *C. glutamicum* strains expressing heterologous PHBHs. White and gray colored bars indicate the concentration of 4-ABA and 4-amino-3-hydroxybenzoic acid (4,3-AHBA), respectively. Data are represented as mean ± standard deviation of biological replicates (*n* = 6). Yellow circles indicate the mean percentage of 4,3-AHBA. The horizontal axis indicates the strains of interest expressing wild type PHBHs. N.D.: not detected

To evaluate the PHBHs under sufficient 4-ABA supply, the strain KC551 (Table 1), which is endowed with an enhanced endogenous 4-ABA biosynthetic pathway, was used as the parental strain for transformation. In KC551, P_*tuf*_ promoters were inserted upstream of the endogenous genes encoding the enzymes of the pentose phosphate pathway, shikimate pathway, subsequent 4-ABA production pathway (*pabABC* operon), and the *ppsA* gene encoding phosphoenolpyruvate synthase, and two additional genes (*qsuB* and endogenous *pobA*) were deleted to prevent unwanted reactions. To confirm the adequate supply of 4-ABA, we measured 4-ABA concentration, glucose concentration, and optical density at 600 nm (OD_600_) every 24 h for 96 h in a deep-well plate culture of KN001 (Fig. 2A), in which the pKCG_P_*tuf*__T1 empty vector was introduced into KC551. The culture was performed using a nutrient-rich CGTG15 medium containing a relatively high concentration of glucose (per liter: 50 g glucose, 10 g tryptone, 20 g (NH_4_)_2_SO_4_, 5 g urea, 1 g KH_2_PO_4_, 1 g K_2_HPO_4_, 0.25 g MgSO_4_·7H_2_O, 10 mg CaCl_2_, 10 mg FeSO_4_·7H_2_O, 10 mg MnSO_4_·5H_2_O, 1 mg ZnSO_4_·7H_2_O, 0.2 mg CuSO_4_·5H_2_O, 0.02 mg NiCl_2_·6H_2_O, 0.2 mg biotin) and supplemented with kanamycin sulfate (50 mg/L). After 96 h of culture, glucose was depleted and OD_600_ reached 40, while 8.8 mM (1.2 g/L) 4-ABA was detected in the culture supernatant, indicating successful overproduction of 4-ABA with an enhanced endogenous biosynthetic pathway.

Subsequently, six strains (KN002–KN007) carrying the plasmids harboring the heterologous *pobA* genes and the control strain KN001 were cultured, and the concentrations of 4-ABA and 4,3-AHBA in the culture supernatants after 86 h were analyzed using high-performance liquid chromatography (HPLC). 4,3-AHBA was detected in the supernatants of all the six strains except in the control strain KN001 (Fig. 2B and Additional file 2: Fig. S1), indicating that the intracellularly expressed heterologous PHBHs could catalyze the 3-hydroxylation of 4-ABA to produce 4,3-AHBA. Additional analysis of cell lysates from KN001–KN007 using sodium dodecyl sulfate-polyacrylamide gel electrophoresis (SDS-PAGE) also revealed additional bands around 40 kDa in all strains except KN001 and KN003 (Additional file 2: Fig. S2), supporting the possibility of heterologous expression. The highest mean 4,3-AHBA concentration (4.5 ± 0.2 mM) and mean 4,3-AHBA percentage (47%) were detected in the culture supernatant of KN003 expressing *Cv*PHBH, which has 61% identity to the amino acid sequence of *Pa*PHBH. Thus, *Cv*PHBH was selected as the wild type enzyme for further amino acid mutagenesis studies.

### Site-directed mutagenesis targeting the active site of *Cv*PHBH

Using *Cv*PHBH as a template enzyme, amino acid mutations effective in increasing the 4,3-AHBA productivity in *C. glutamicum* were investigated using site-directed mutagenesis of active site residues.

**Fig. 3:**
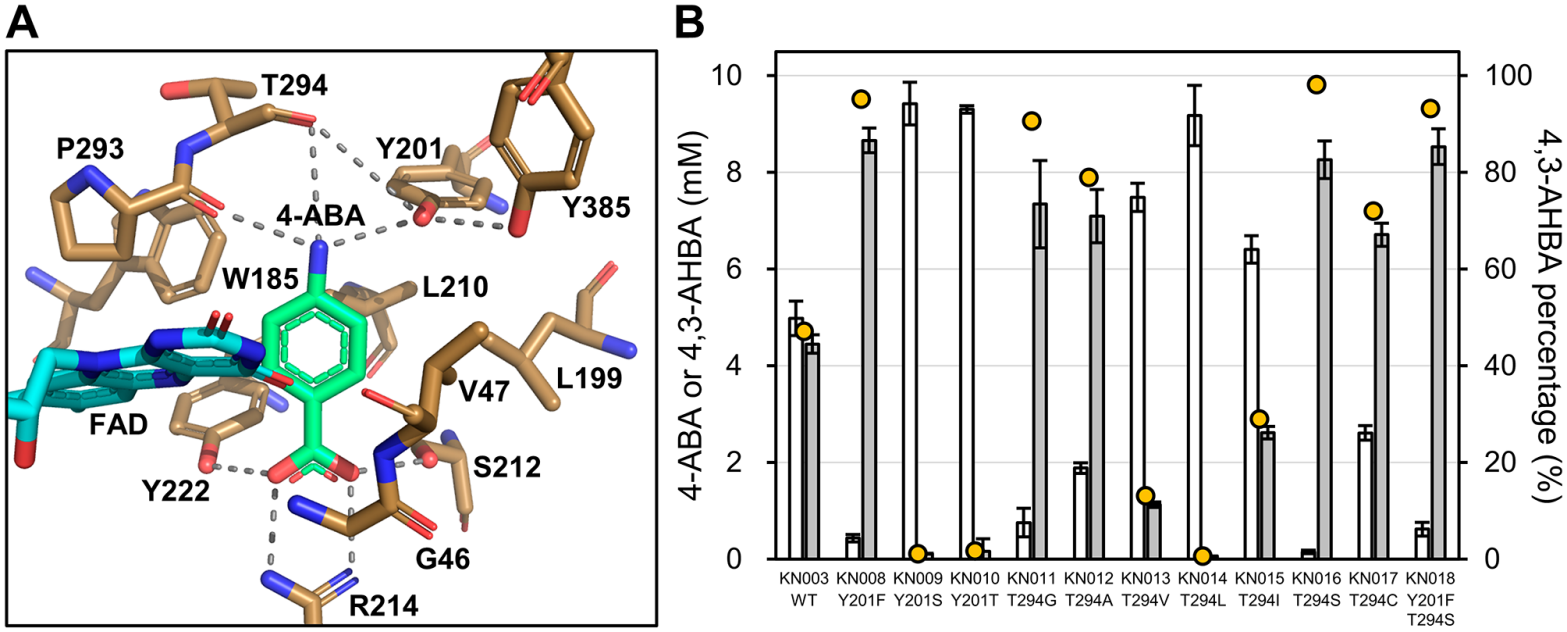
Effect of site-directed mutations in the active site of *Cv*PHBH on 4-amino-3-hydroxybenzoic acid (4,3-AHBA) productivity of *Corynebacterium glutamicum*. **A** Three-dimensional structural model of *Cv*PHBH. The brown stick represents the selected residues in the active site. The green stick represents 4-aminobenzoic acid (4-ABA). The sky-blue stick represents FAD. The dotted line represents hydrogen bonds. The molecular structure model was visualized using the PyMOL Molecular Graphics System (Version 2.0, Schrödinger, LLC, New York, NY, USA). **B** The results of HPLC analysis of the culture supernatants of *C. glutamicum* strains expressing site-directed mutants of *Cv*PHBH. White and gray colored bars indicate the concentration of 4-ABA and 4,3-AHBA, respectively. Data are represented as mean ± standard deviation of biological replicates (*n* = 6). Yellow circles indicate the mean percentage of 4,3-AHBA. The horizontal axis indicates the strains of interest expressing *Cv*PHBH mutants. WT: wild type

PHBH is classified as a flavoprotein monooxygenase (FPMO) and normally catalyzes the 3-hydroxylation of 4-HBA to PCA using reduced nicotinamide adenine dinucleotide phosphate (NADPH), molecular oxygen (O_2_), and flavin adenine dinucleotide (FAD) [16]. Based on a three-dimensional structure model of *Cv*PHBH constructed using homology modeling, 11 residues (G46, V47, W185, L199, Y201, L210, S212, R214, Y222, P293, and T294) located within 4 Å of 4-ABA and one residue (Y385) involved in the formation of hydrogen-bond networks between the substrate and the protein surface [16] are shown in Fig. 3A. All 12 of these amino acid residues were identical to the residues at the same position in the amino acid sequence of *Pa*PHBH (Additional file 2: Fig. S3). Among them, in light of the studies on *Pa*PHBH and *Pf*PHBH [16, 35–41], three residues (S212, R214, and Y222) that could form noncovalent bonds with the carboxy group of 4-ABA and another three (Y201, P293, and T294) that could form a hydrogen-bond loop with the amine group of 4-ABA were considered to be largely responsible for the 3-hydroxylation activity of *Cv*PHBH. However, because both 4-ABA and 4-HBA have the same structure on the carboxy side, the effect of mutagenesis on the carboxy side residues in improving the activity toward 4-ABA was considered to be relatively small. In contrast, because deprotonation of the phenolic group of 4-HBA is an important step in the catalytic process of *Pa*PHBH [16], Y201 was deemed a pivotal residue, as the phenolic group of the side chain could form hydrogen bonds with the amino group of 4-ABA. Furthermore, T294 was considered a suitable residue to modify the activity of *Cv*PHBH by mutagenesis while preserving the enzyme function, because the carbonyl group of the main chain, whose molecular species is unaffected by mutagenesis, could be involved in the formation of hydrogen bonds with Y201 and 4-ABA. Indeed, mutations of T294 have been reported in *Pa*PHBH mutants with increased hydroxylation activity toward PCA [38–40]. On the contrary, mutations of P293, which has been reported to be responsible for the movement of FAD in the catalytic cycle [41], were expected to disrupt the catalytic function of the enzyme. Therefore, we focused on Y201 and T294 as target residues for site-directed mutagenesis and screened 10 mutants (*Cv*PHBH^Y201F^, *Cv*PHBH^Y201S^, *Cv*PHBH^Y201T^, *Cv*PHBH^T294G^, *Cv*PHBH^T294A^, *Cv*PHBH^T294V^, *Cv*PHBH^T294L^, *Cv*PHBH^T294I^, *Cv*PHBH^T294S^, and *Cv*PHBH^T294C^), excluding mutations that were expected to disrupt the catalytic function beforehand.

The *C. glutamicum* strains expressing the selected *Cv*PHBH mutants (KN008–KN017) were constructed, and the concentrations of 4,3-AHBA and 4-ABA in the culture supernatants of these strains after 86 h were analyzed. The results showed an improvement in the mean 4,3-AHBA concentration and mean 4,3-AHBA percentage when five single-mutants (*Cv*PHBH^Y201F^, *Cv*PHBH^T294G^, *Cv*PHBH^T294A^, *Cv*PHBH^T294S^, and *Cv*PHBH^T294C^) were expressed (Fig. 3B). Conversely, expression of five single-mutants (*Cv*PHBH^Y201S^, *Cv*PHBH^Y201T^, *Cv*PHBH^T294V^, *Cv*PHBH^T294L^, and *Cv*PHBH^T294I^) did not increase the 4,3-AHBA productivity compared with the productivity in the wild type *Cv*PHBH. The highest mean 4,3-AHBA concentration was detected in KN008 expressing *Cv*PHBH^Y201F^ (8.7 ± 0.3 mM, 95%), whereas the highest mean 4,3-AHBA percentage was detected in KN016 expressing *Cv*PHBH^T294S^ (8.3 ± 0.4 mM, 98%).

Following a marked increase in the 4,3-AHBA productivity in strains expressing *Cv*PHBH^Y201F^ and *Cv*PHBH^T294S^, we evaluated the 4,3-AHBA productivity of KN018 expressing the double-mutant *Cv*PHBH^Y201F/T294S^. KN018 exhibited a higher mean 4,3-AHBA concentration (8.5 ± 0.4 mM) and mean 4,3-AHBA percentage (93%) than did KN003 expressing the wild type *Cv*PHBH; however, the improvement of mean 4,3-AHBA percentage was not as remarkable as that observed when the respective single-mutants were expressed.

### Random mutagenesis screening with colorimetric detection of 4,3-AHBA

In parallel with site-directed mutagenesis, a random library of *Cv*PHBH mutants was screened using a newly constructed colorimetric assay method that estimates the concentration of 4,3-AHBA in culture supernatants. This was done because we believe that random mutagenesis of the entire gene sequence can complement the search for effective mutations at positions other than the active site, which are relatively difficult to find using rational design based on the three-dimensional structure of the enzyme.

**Fig. 4:**
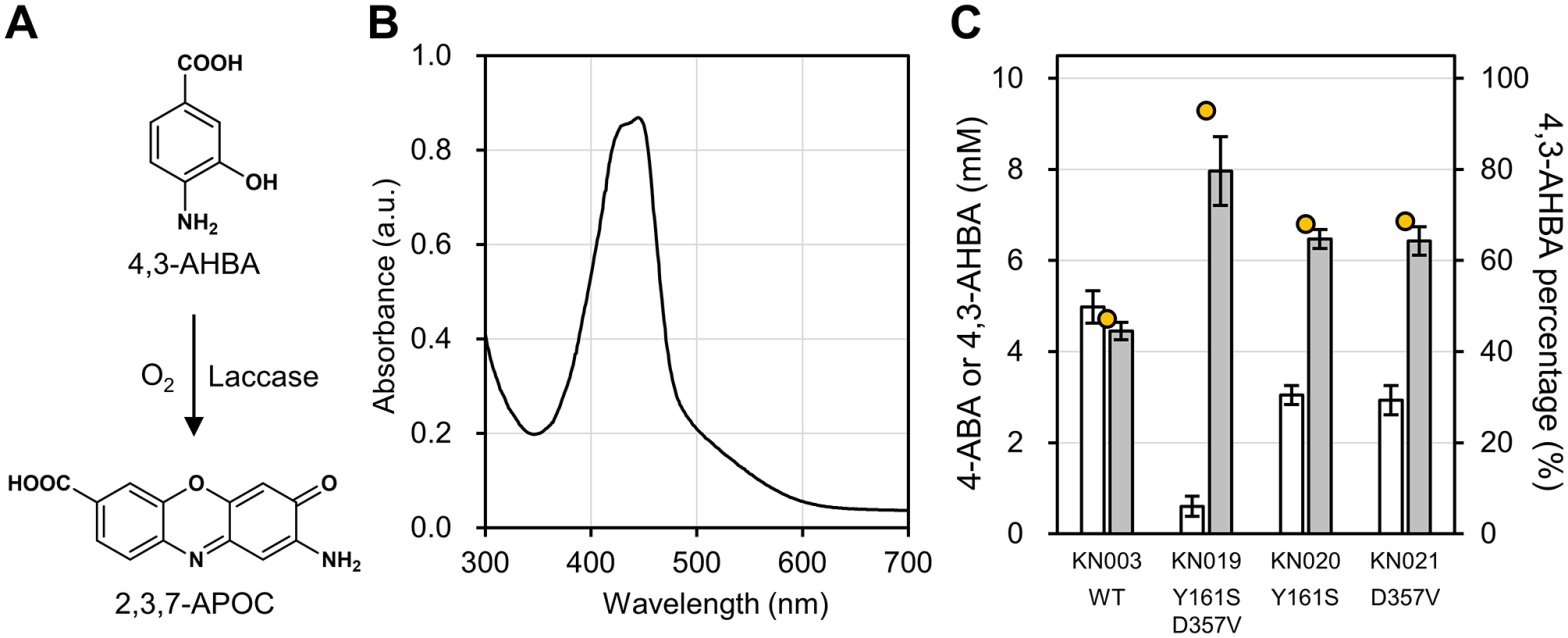
Screening of the *Cv*PHBH random mutant library using the laccase-mediated colorimetric method. **A** Schematic representation of the laccase-catalyzed oxidative dimerization of 4-amino-3-hydroxybenzoic acid (4,3-AHBA) to 2-aminophenoxazin-3-one-7-carboxylic acid (2,3,7-APOC). **B** The absorption spectrum of an orange-yellow reaction solution after mixing laccase and 4,3-AHBA in 100 mM citrate buffer (pH 4.5). **C** The results of HPLC analysis of the culture supernatants of *Corynebacterium glutamicum* strains expressing the *Cv*PHBH mutants found using the random mutagenesis screening. White and gray colored bars indicate the concentration of 4-aminobenzoic acid (4-ABA) and 4,3-AHBA, respectively. Data are represented as mean ± standard deviation of biological replicates (*n* = 6). Yellow circles indicate the mean percentage of 4,3-AHBA. The horizontal axis indicates the strains of interest expressing *Cv*PHBH mutants. WT: wild type

For rapid screening, we first established a method to estimate the concentration of 4,3-AHBA by oxidizing 4,3-AHBA with a commercially available laccase and spectrophotometrically measuring the amount of dye produced (Fig. 4A). Fortunately, it has been reported that oxidative dimerization of 4,3-AHBA occurs naturally under aerobic conditions at room temperature to form 2-aminophenoxazin-3-one-7-carboxylic acid (2,3,7-APOC), which is easily observed as orange-yellow with the naked eye [42]. In addition, studies on multi-copper oxidases, such as tyrosinase and laccase, have shown that these enzymes oxidize molecules retaining *ortho*-aminophenol group with O_2_ and the subsequent nonenzymatic dimerization reaction produces phenoxazinone dyes [43, 44]. Given the commercial availability of laccases, the colorimetric assay method based on the oxidation of 4,3-AHBA with laccase was developed. The addition of 4,3-AHBA to 0.1 M citrate buffer (pH 4.5) containing the laccase caused an immediate change in the color of the solution, which turned orange-yellow. The absorption spectrum of the reaction solution showed an absorption peak at ca. 446 nm (Fig. 4B), which was consistent with previously reported results [42]. In addition, the *m/z* of the eluate with an absorption peak at ca. 446 nm in the liquid chromatography-mass spectrometry (LC-MS) measurement was 257 (Additional file 2: Fig. S4), which was consistent with that of 2,3,7-APOC ([M + H]^+^ = 257).

A plasmid library harboring the genes encoding random *Cv*PHBH mutants in the expression cassette of pKCG_P_*tuf*__T1 was then generated using error-prone PCR and used to transform KC551. The individual culture supernatants of the resulting 1152 transformant colonies were each mixed with the laccase solution and assayed by absorbance at 446 nm. Sequencing of the *Cv*PHBH mutant expressed in the strain with the highest absorbance resulted in the identification of the *Cv*PHBH^Y161S/D357V^ double-mutant. The effect of the double-mutation, together with the effect of the two single mutations that comprise it, on the 4,3-AHBA productivity was re-evaluated using HPLC (Fig. 4C). The analysis of the culture supernatant of KN019 expressing *Cv*PHBH^Y161S/D357V^ after 86 h revealed a mean 4,3-AHBA concentration of 8.0 ± 0.8 mM and a mean percentage of 93%. On the contrary, when KN020 and KN021 expressing *Cv*PHBH^Y161S^ and *Cv*PHBH^D357V^ were used, the 4,3-AHBA productivity was 6.5 ± 0.2 mM (68%) and 6.4 ± 0.3 mM (69%), respectively.

### Exploration of effective mutations around FAD

**Fig. 5:**
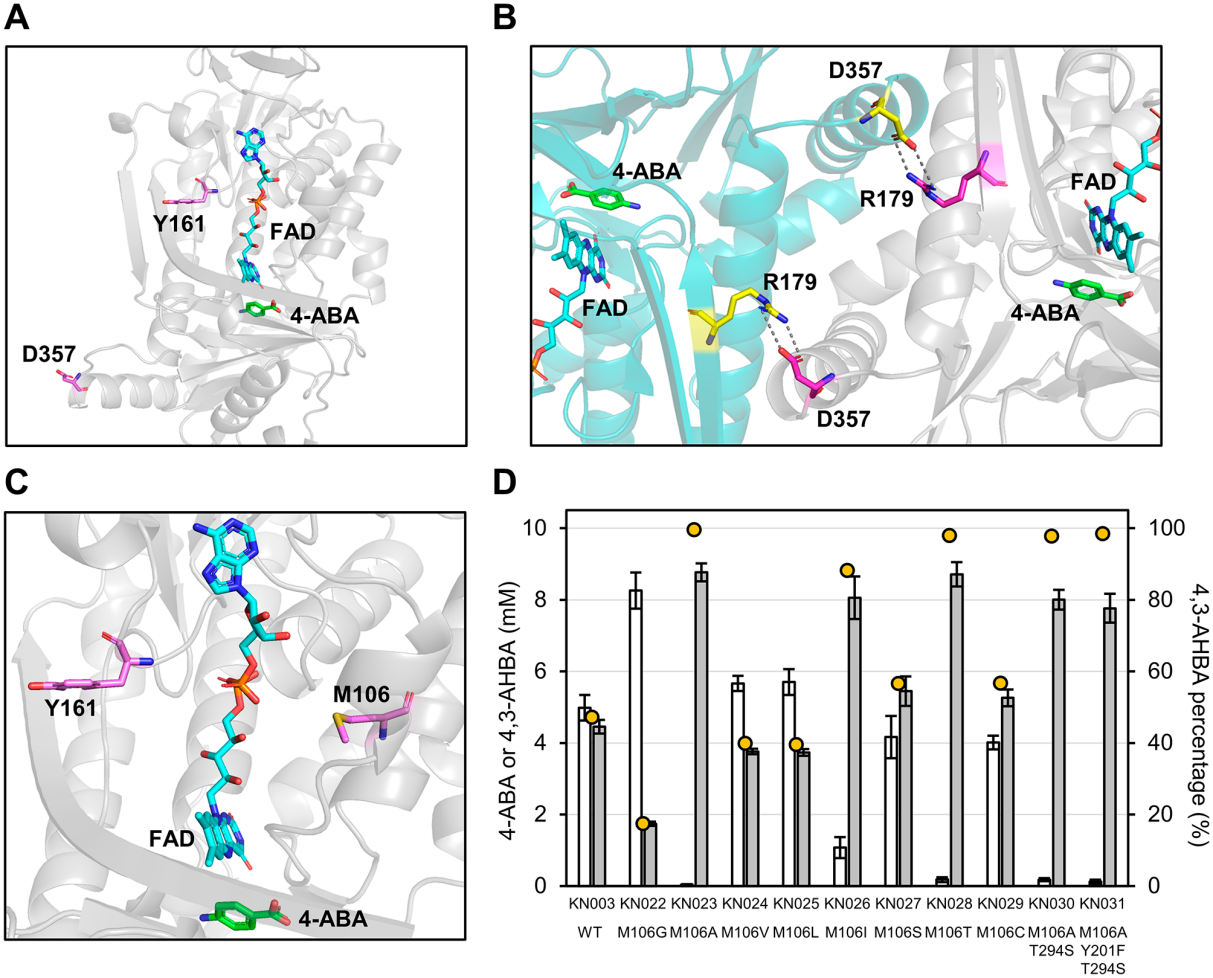
Site-directed mutagenesis on the M106 residue of the *Cv*PHBH. The molecular structure model was visualized using the PyMOL Molecular Graphics System (Version 2.0, Schrödinger, LLC, New York, NY, USA). **A** The location of Y161 and D357 in the three-dimensional structure model of *Cv*PHBH. Purple stick represents the Y161 and D357 residues. Green and sky-blue stick represents 4-aminobenzoic acid (4-ABA) and FAD, respectively. **B** Interaction of D357 and R179 in the crystal structure of *Pa*PHBH (PDB ID: 1IUT). Purple and yellow sticks represent the designated amino acids of chain A and chain B, respectively. The dotted line represents hydrogen bonds. **C** The location of M106 in the three-dimensional structure. Purple stick represents the Y161 and M106 residues. **D** The results of HPLC analysis of the culture supernatants of *Corynebacterium glutamicum* strains expressing the *Cv*PHBH mutants. White and gray colored bars indicate the concentration of 4-ABA and 4-amino-3-hydroxybenzoic acid (4,3-AHBA), respectively. Data are represented as mean ± standard deviation of biological replicates (*n* = 6). Yellow circles indicate the mean percentage of 4,3-AHBA. The horizontal axis indicates the strains of interest expressing *Cv*PHBH mutants. WT: wild type

With regard to the two mutation sites found in the random mutagenesis screening, Y161 was located around FAD, a cofactor required in the catalytic cycle, and D357 was located on the surface of the enzyme in the three-dimensional structure model of *Cv*PHBH (Fig. 5A). Studies on *Pf*PHBH have reported that F161 is involved in the binding of FAD and NADPH [45, 46]. D357 was also found to interact with R179 of the other polypeptide chain in the crystal structure of *Pa*PHBH (Fig. 5B), which is known to form a homodimer [16], indicating that D357 contributes to the formation of the homodimer structure. Because Y161 is located close to FAD, we were interested in introducing mutations into the M106 residue, which is opposite to Y161 across FAD (Fig. 5C) and was expected to affect the position and movement of the cofactor. Therefore, eight strains (KN022–KN029) expressing *Cv*PHBH mutants with a seemingly nondestructive mutations introduced at the M106 residue (*Cv*PHBH^M106G^, *Cv*PHBH^M106A^, *Cv*PHBH^M106V^, *Cv*PHBH^M106L^, *Cv*PHBH^M106I^, *Cv*PHBH^M106S^, *Cv*PHBH^M106T^, *Cv*PHBH^M106C^) were constructed and evaluated for the 4,3-AHBA productivity (Fig. 5D). Unexpectedly, KN023 expressing *Cv*PHBH^M106A^ showed even higher 4,3-AHBA productivity (8.8 ± 0.3 mM, 99%) than did KN008 and KN016 expressing the active site mutants *Cv*PHBH^Y201F^ and *Cv*PHBH^T294S^. Furthermore, evaluation of KN030 and KN031, expressing a double-mutant *Cv*PHBH^M106A/T294S^ and a triple-mutant *Cv*PHBH^M106A/Y201F/T294S^, respectively, also resulted in high 4,3-AHBA productivity (8.0 ± 0.3 mM, 98%; 7.8 ± 0.4 mM, 98%), indicating that the M106A mutation located around FAD is compatible with the Y201F and T294S mutations located at the active site.

### Production of 4,3-AHBA in fed-batch culture

**Fig. 6:**
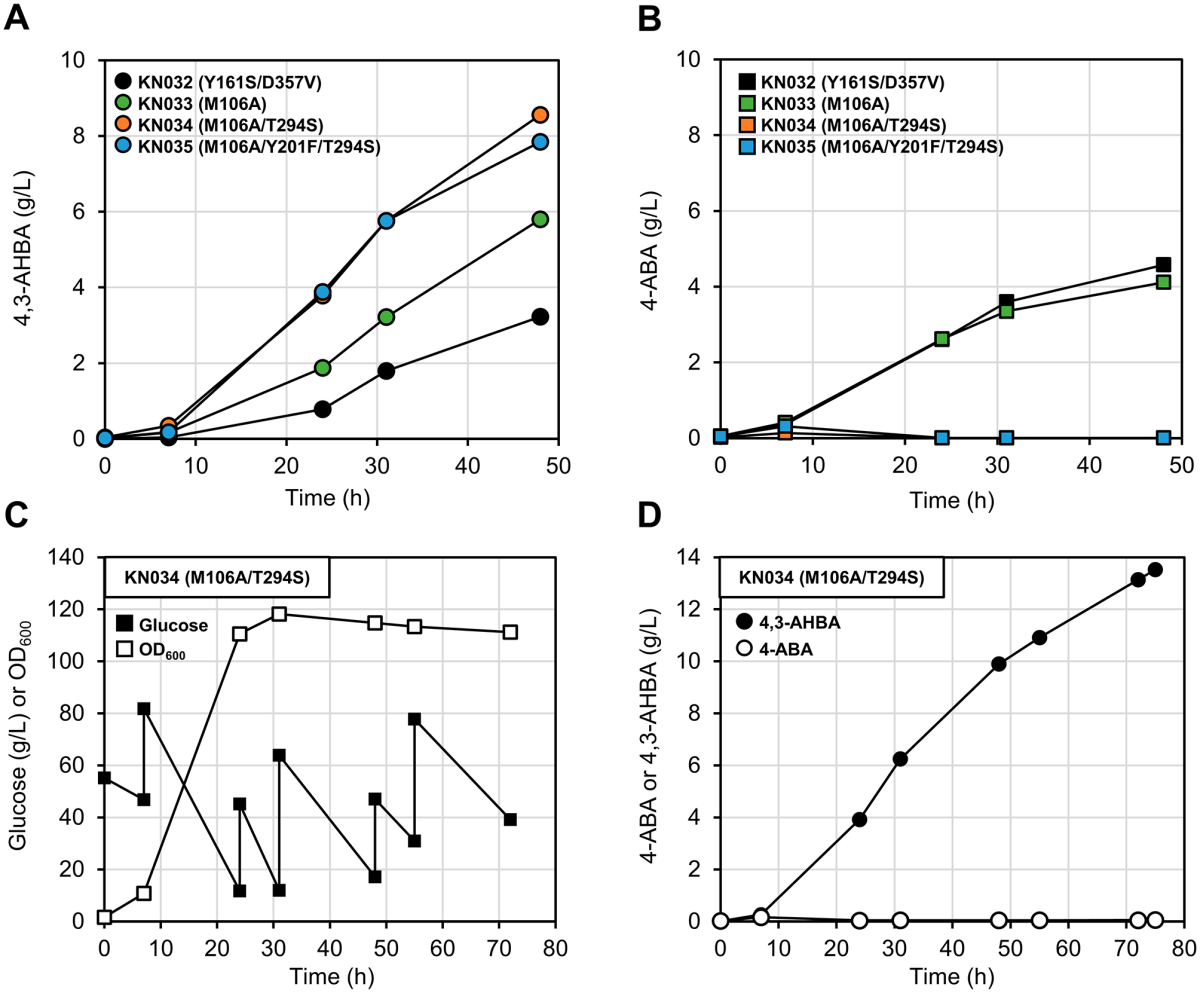
Production of 4-amino-3-hydroxybenzoic acid (4,3-AHBA) in fed-batch culture with nutrient-rich CGTG15 medium using *Corynebacterium glutamicum* strains expressing the selected *Cv*PHBH mutants. **A, B** Time course of 4,3-AHBA and 4-aminobenzoic acid (4-ABA) concentrations in culture supernatants. The color of circles and squares indicates the following strains: black, KN032 (*Cv*PHBH^Y161S/D357V^); green, KN033 (*Cv*PHBH^M106A^); orange, KN034 (*Cv*PHBH^M106A/T294S^); and blue, KN035 (*Cv*PHBH^M106A/Y201F/T294S^). **C, D** Fed-batch culture of KN034 performed for longer duration. Each plot indicates: open square, OD_600_; filled square, glucose concentration; open circle, 4-ABA concentration; filled circle, 4,3-AHBA concentration. Time variations of fermentation process parameters are shown in Additional file 2: Fig. S6

To investigate their ability to produce 4,3-AHBA on a gram-scale, *C. glutamicum* strains expressing *Cv*PHBH mutants were grown in fed-batch culture. Preliminary culture of *C. glutamicum* NBRC 12168 in nutrient-rich CGTG15 medium supplemented with 100 mM (15.3 g/L) 4,3-AHBA resulted in sufficient growth (Additional file 2: Fig. S5), indicating that *C. glutamicum* is tolerant to high concentrations of 4,3-AHBA. To further enhance the supply of 4-ABA, *aroG*^*D146N*^ from *E. coli* and *aroF*^*P155L*^ from *C. glutamicum* that encode 3-deoxy-D-arabino-heptulosonate-7-phosphate (DAHP) synthase with feedback-resistance [47, 48] were introduced into KC551; the resulting strain KC617 was transformed with plasmids harboring the genes encoding the selected *Cv*PHBH mutants (*Cv*PHBH^Y161S/D357V^, *Cv*PHBH^M106A^, *Cv*PHBH^M106A/T294S^, and *Cv*PHBH^M106A/Y201F/T294S^), resulting in strains KN032–KN035. Comparison of these strains after 48 h of fed-batch culture in nutrient-rich CGTG15 medium revealed the highest 4,3-AHBA productivity in KN034 expressing *Cv*PHBH^M106A/T294S^ (Fig. 6A and B). Therefore, we again performed a longer culture with KN034. The fed-batch culture of KN034 provided glucose under constant pH, temperature, and dissolved O_2_ conditions with an initial culture volume of 60 mL using nutrient-rich CGTG15 medium (Fig. 6C and Additional file 2: Fig. S6). After 75 h of culture (final volume, 84 mL), the concentration of 4,3-AHBA in the culture supernatant reached 13.5 g/L (88 mM; Fig. 6D), and that of 4-ABA was 0.059 g/L (0.43 mM). The yield of 4,3-AHBA per molecule of glucose consumed was calculated to be 0.072 g/g_glucose_.

### Calculation of carbon yield for 4,3-AHBA production

**Fig. 7:**
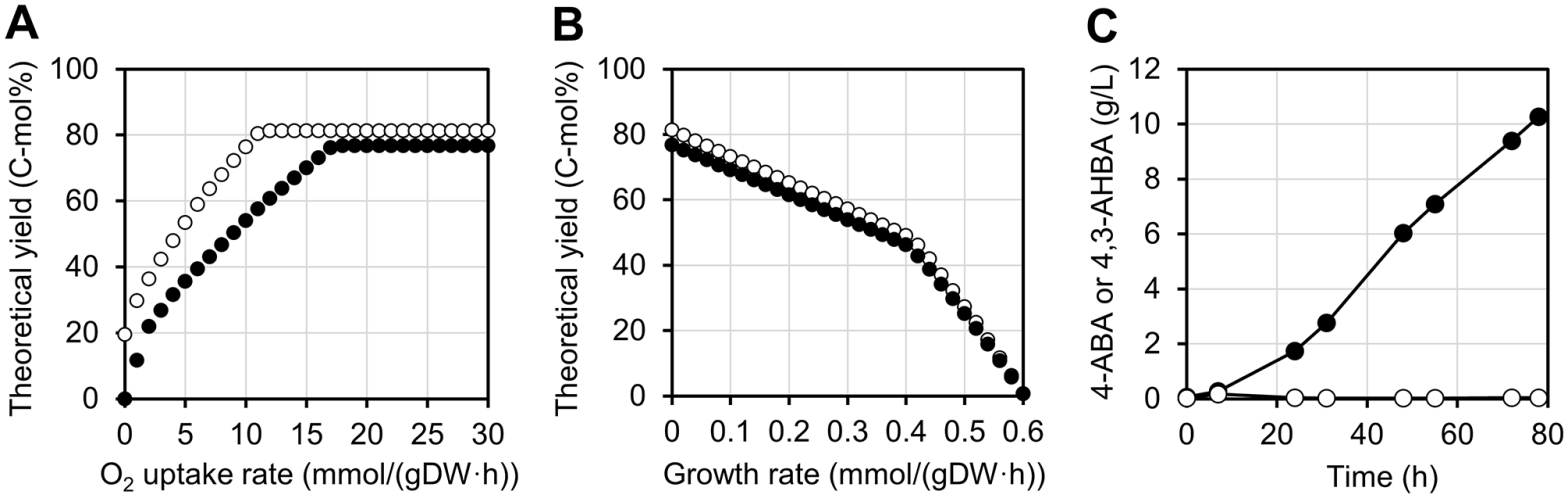
*In silico* simulation and fed-batch culture for calculation of the carbon yield. Open and filled circles indicate 4-aminobenzoic acid (4-ABA) and 4-amino-3-hydroxybenzoic acid (4,3-AHBA) production, respectively. **A** The theoretical yield calculated from each individual flux balance analysis (FBA) was plotted as the O_2_ uptake rate was varied between 0 and 30 mmol/(gDW·h). The growth rate was fixed at 0 mmol/(gDW·h). **B** The theoretical yield calculated from each individual FBA was plotted as the growth rate was varied between 0 and 0.6 mmol/(gDW·h). The O_2_ uptake rate was fixed at 20 mmol/(gDW·h). **C** The fed-batch culture of KN034 expressing *Cv*PHBH^M106A/T294S^ in chemically defined CGXII medium

To estimate the possibility for improvement in the 4,3-AHBA productivity, the theoretical maximum yields of 4-ABA and 4,3-AHBA were calculated by performing flux balance analysis (FBA) using a model constructed by adding heterologous 4,3-AHBA production reactions to *i*CGB21FR [49], a genome-scale metabolic model of *C. glutamicum*. Because *i*CGB21FR originally included the 4-ABA production reaction as part of the folate synthesis pathway, the following five additional reactions were added to the model: 3-hydroxylation (4-ABA + O_2_ + NADPH + H^+^ = 4,3-AHBA + H_2_O + NADP^+^), extracellular transport reactions of 4-ABA and 4,3-AHBA, and sink reactions of 4-ABA and 4,3-AHBA. The objective reaction of the FBA was set to maximize the sink reactions of the corresponding products. The uptake rates of glucose, PCA, and urea were fixed at 10, 0, and 0 mmol/(gDW·h), respectively, in all simulations to set glucose as the sole carbon source. The theoretical yield was expressed in terms of C-mol%, based on the value calculated by dividing the corresponding production rate by the glucose uptake rate. The theoretical maximum yields were calculated to be 81.3 C-mol% (0.53 g/g_glucose_) for 4-ABA and 76.8 C-mol% (0.56 g/g_glucose_) for 4,3-AHBA when the O_2_ uptake rate and growth rate were set to 20 and 0 mmol/(gDW·h), respectively (Fig. 7A and B). The theoretical maximum yield to produce 4-ABA (81.3 C-mol%) was consistent with that reported previously [50]. The fed-batch culture of KN034 in the chemically defined CGXII medium [51–53] yielded 10.3 g/L (67 mM) 4,3-AHBA (Fig. 7C), corresponding to 0.071 g/g_glucose_ and 9.8 C-mol%. This yield was 12.8% of the theoretical maximum yield (76.8 C-mol%), which suggests that there is a lot of room for future improvement.

## Discussion

The design and implementation of artificial metabolic pathways for production of aromatic chemicals from renewable resources are important in providing support for future expansion of renewable chemical materials. The pathway designed in this study to produce the PBO precursor, 4,3-AHBA, from glucose in gram-scale production was realized by applying the newly explored 3-hydroxylation of 4-ABA using the expression of *Cv*PHBH and its mutants in *C. glutamicum*. Random mutagenesis screening using the laccase-mediated colorimetric assay was also useful in the search for effective mutations located outside the active site of *Cv*PHBH.

In the mutagenesis study, three single mutations at different residues, namely Y201F, T294S, and M106A, were identified to be responsible for marked improvement in the 4,3-AHBA productivity (Figs. 3B and 5D). One reason for the enhanced 4,3-AHBA productivity following the Y201F mutation may be the loss of the hydrogen bond between the phenolic group on the side chain and the amine group of 4-ABA. These results are consistent with the report on the enhanced hydroxylation activity toward 4-aminophenylacetic acid (4-APA) by the S146A mutation in 4-hydroxyphenylacetate 3-hydroxylase (EC 1.14.14.9) from *Acinetobacter baumannii* (*Ab*HPAH), which belongs to a different group of FPMOs [54]. Dhammaraj et al. proposed that the interaction between the hydroxy group of S146 and the phenolic group of 4-hydroxyphenylacetic acid (4-HPA) in the active site of *Ab*HPAH contributes to substrate recognition and that the S146A mutation removes this interaction, allowing the enzyme to recognize its non-natural substrate, 4-APA, similarly to its natural substrate, 4-HPA [54]. Thus, the alteration in substrate recognition by *Cv*PHBH^Y201F^ due to the removal of the hydrogen bond between Y201 and 4-ABA may have contributed to the increased productivity of 4,3-AHBA. However, removal of the phenolic group at position Y201 does not appear to be essential for efficient 3-hydroxylation of 4-ABA by *Cv*PHBH, as indicated by the finding of highly active mutants, such as *Cv*PHBH^T294S^ and *Cv*PHBH^M106A^, that did not contain the Y201F mutation. In particular, the T294S mutation of the T294 residue, whose main chain carbonyl group could form a hydrogen bond with substrates and Y201 (Fig. 3A), increased the 4,3-AHBA productivity, although the nature of the molecular environment that directly interacts with 4-ABA is unlikely to change because the side chain of the T294 residue faces away from 4-ABA. It was also observed that mutations to sterically smaller amino acids, such as Gly, Ala, Ser, and Cys, at position T294 tended to increase the 4,3-AHBA productivity (Fig. 3B). Previously, Chen et al. reported that introduction of the T294A mutation effectively improved the reactivity of *Pa*PHBH toward PCA. They proposed that the increased flexibility of the hydrogen-bond loop due to the disruption of the hydrogen bond between T294 and T347 contributed to this improvement [38]. Our results are not consistent with this proposal, as 4,3-AHBA productivity was increased even for mutations, such as T294S, which do not appear to disrupt the hydrogen bond with T347. However, because mutation to sterically smaller amino acids is believed to create conformational space and increase the flexibility of the residue, the effect of increasing the flexibility of the hydrogen-bond loop would be expected for the T294S mutation in *Cv*PHBH as well as for the T294A mutation in *Pa*PHBH. Therefore, a possible explanation for the effectiveness of the mutation at position T294 in improving the 4,3-AHBA productivity is the increased flexibility of the hydrogen-bond loop at the active site of *Cv*PHBH. Although it is not clear how the increased flexibility of the hydrogen-bond loop correlated with the increased enzymatic activity in our case, it is possible that the increased movement of the main chain of T294 improved the probability that 4-ABA, the carbonyl group of T294 and P293, the phenolic group of Y201, and FAD would be aligned in an appropriate position relative to each other in the catalytic cycle. Regarding the M106A mutation, it was surprising that the introduction of a single mutation at the M106 residue, which is located away from the active site, dramatically improved the 4,3-AHBA productivity (Fig. 5D). Although the reason for the increased productivity with the M106A mutation cannot be fully interpreted from the results of this study, given that FAD exists in multiple conformations in the catalytic cycle [37, 41], the steric reduction of the M106 position may be a contributing factor to improve the movement of FAD and enzyme activity. In addition, the 4,3-AHBA productivity of KN034 expressing *Cv*PHBH^M106A/T294S^ was superior to that of KN033 expressing *Cv*PHBH^M106A^ in the fed-batch culture (Fig. 6A), which indicates that the two beneficial single mutations, M106A and T294S, could be combined without mutual interference. It should be noted, however, that the comparisons made in this study regarding PHBHs were based on the concentration of 4,3-AHBA produced in the culture supernatant of *C. glutamicum* strains that express PHBHs intracellularly. Further detailed biochemical analysis of *Cv*PHBH and its mutants will provide a better understanding of the effects of mutations on the substrate specificity of *Cv*PHBH and on the 4,3-AHBA productivity of *C. glutamicum* when the *Cv*PHBH mutants are expressed.

FPMOs are believed to have wide applications as biocatalysts for the synthesis of value-added chemicals because they can catalyze various regioselective monooxygenation reactions, such as hydroxylation, Baeyer–Villiger oxidation, and epoxidation [55, 56]. Currently, several enzymes classified as FPMOs are known to perform *ortho*-hydroxylation of aniline derivatives, such as kynurenine 3-monooxygenase (EC 1.14.13.9), which catalyzes the hydroxylation of kynurenine using O_2_ and NADPH as cosubstrates to yield 3-hydroxykynurenine in the *L*-tryptophan degradation pathway [57], and anthranilate 3-monooxygenase (EC 1.14.14.8), which converts anthranilate to 3-hydroxyanthranilate in the anthranilate degradation pathway [58]. The findings for *Cv*PHBH and its mutants would be new examples of FPMOs catalyzing the *ortho*-hydroxylation of aniline derivatives and would contribute to our understanding of this group of enzymes, leading to the expansion of their application.

From a practical point of view, there is a huge scope for further improving the productivity and purity of 4,3-AHBA using metabolic and culture engineering technologies. It has been reported that the introduction of heterologous *pabAB* and *pabC* genes encoding ADC synthase and ADC lyase contributed to the improvement of 4-ABA productivity [17], which can be employed to further increase the supply of intracellular 4-ABA. In addition, because PHBHs consume O_2_ and NADPH simultaneously during their reaction, controlling the dissolved O_2_ content during culture and providing sufficient NADPH by altering the metabolic redox balance in the cell could increase the productivity. The genome-scale metabolic model constructed in this study will be helpful in effectively formulating strategies for future strain design to produce 4,3-AHBA. Furthermore, the purity and cost of downstream processing can be improved by controlling the oxidative coloration of the culture supernatant. We expect that these improvements will enable the production of 4,3-AHBA from biomass resources at an industrially feasible cost for practical application.

## Conclusions

Expression of the *Cv*PHBH mutants in the industrially relevant bacterium, *C. glutamicum*, enabled efficient 3-hydroxylation of 4-ABA derived from the shikimate pathway, leading to *de novo* production of 4,3-AHBA from glucose. The fed-batch culture of *C. glutamicum* strain KN034 expressing *Cv*PHBH^M106A/T294S^ with enhanced upstream pathway yielded 13.5 g/L (0.072 g/g_glucose_) 4,3-AHBA in nutrient-rich medium. The carbon yield (9.8 C-mol%) of the KN034 culture in chemically defined CGXII medium was 12.8% of the theoretical maximum yield (76.8 C-mol%) simulated with a genome-scale metabolic model. The *Cv*PHBH mutants obtained in this study are useful for elucidating the molecular mechanism of regioselective monooxygenation of the aniline moiety. These findings contribute to the diversification of aromatic chemicals derived from biomass resources to address the global demand for sustainable chemical products.

## Methods

### Chemicals and reagents

Standard chemicals for the analysis of 4-ABA and 4,3-AHBA were purchased from Tokyo Chemical Industry (Tokyo, Japan). All other chemicals were purchased from FUJIFILM Wako Pure Chemical Corporation (Osaka, Japan). DNA synthesis and sequencing were performed by Eurofins Genomics (Tokyo, Japan). DNA amplification was performed using KOD One® Master Mix (TOYOBO, Tokyo, Japan), PrimeSTAR® Mutagenesis Basal Kit (Takara Bio, Shiga, Japan), or Diversify™ PCR Random Mutagenesis Kit (Takara Bio). The pHSG299 plasmid, *Dpn*I, NucleoSpin® PCR and Gel Clean-up, In-Fusion® HD Cloning Kit, and NucleoSpin® Plasmid EasyPure were purchased from Takara Bio and used for DNA manipulation. The pHM1519 plasmid was extracted from *C. glutamicum* NBRC 12169 [59]. *ECOS*™ Competent *E. coli* JM109 (NIPPON GENE, Tokyo, Japan) was used for plasmid preparation. Difco™ Terrific Broth and Difco™ LB Broth were purchased from Becton, Dickinson and Company (Franklin Lakes, NJ, USA) and supplemented as appropriate with 1.5% agar and 50 mg/L kanamycin sulfate for culturing *E. coli* strains. Bacto™ Tryptone was purchased from Life Technologies Corporation (Waltham, MA, USA). Mini-PROTEAN® TGX™ Stain-Free Gels (Any kD, 15-well; Bio-Rad Laboratories, Hercules, CA, USA), Precision Plus Protein™ Unstained Standards (Bio-Rad Laboratories), and 3X SDS-PAGE Loading Buffer (BioVision, Milpitas, CA, USA) were used for SDS-PAGE analysis.

### Construction of plasmids and bacterial strains

The *C. glutamicum* strains used in the study are listed in Table 1. The plasmids and primers used in the study are listed in Additional file 1: Table S2 and S3, respectively. The genomic DNA sequence and gene numbers for *C. glutamicum* were referred from previous reports [60, 61]. The nucleotide sequences of the *pobA* genes codon-optimized for *C. glutamicum* are listed in Additional file 3. Each codon-optimized *pobA* gene was amplified from synthetic DNA using the appropriate primer pair and assembled into the pKCG_P_*tuf*__T1 vector. Plasmids for expression of site-directed PHBH mutants and *aroF*^*P155L*^ were constructed using appropriate overlapping primer pairs. The random mutant library was generated by assembling the DNA fragment of the *pobA* gene for *Cv*PHBH amplified with error-prone PCR and the linearized pKCG_P_*tuf*__T1 vector. The error-prone PCR was performed in buffer condition 5 (nine mutations per 1000 bp) according to the kit instructions. The recombinant *C. glutamicum* strains were constructed from *C. glutamicum* NBRC 12168 using a two-step homologous recombination system with a suicide vector containing the *sacB* gene from *Bacillus subtilis* [62, 63]. The *C. glutamicum* strains used to evaluate the 4,3-AHBA productivity were constructed by incorporating the appropriate plasmids into KC551 or KC617. The *C. glutamicum* strains were transformed using electroporation [64] with an ELEPO21 (Nepa Gene, Chiba, Japan) under the following conditions: poring pulse (1400 V voltage, 3.5 ms pulse length, 50 ms pulse interval, 1 number of pulses, + polarity) and transfer pulse (200 V voltage, 50 ms pulse length, 50 ms pulse interval, 3 number of pulses, ± polarity).

### Growth conditions of *C. glutamicum* strains

Single colonies of positive transformants were grown aerobically in nutrient-rich CGTG15 medium supplemented with kanamycin sulfate (50 mg/L). The composition of CGTG15 medium was designed with reference to CGXII medium [51–53]. Transformants of KC551 were cultured at 30 °C and 800 rpm in 750 µL of the medium in deep-well plates sealed with Axygen® Breathable Sealing Film (Corning Inc., Corning, NY, USA) using M·BR-034P shaker (TAITEC Corporation, Saitama, Japan). To produce 4,3-AHBA in fed-batch culture, the strains were precultured in 6 mL of the medium in a test tube for 24 h at 30 °C. Thereafter, 3 mL of the precultured cells were used to inoculate 60 mL of the medium containing 250 mg/L antifoam FERMOL1000 (Kao Corporation, Tokyo, Japan) in a small-scale multi-channel fermenter (Bio Jr.8; ABLE, Tokyo, Japan), and cultured at 32 °C (aeration, 60 mL/min). Agitation was automatically controlled to maintain the dissolved O_2_ concentration constant at 0.2 ppm. The pH was maintained at 7.2 by automatic addition of 10% ammonia solution, and glucose in the culture medium was supplemented with 60% (w/w) glucose solution as needed to prevent depletion. Aliquots of culture medium were taken to calculate cell density, followed by quantification of products and glucose. Cell density was calculated by monitoring the OD_600_ using a spectrophotometer.

### SDS-PAGE analysis

Aliquots (60 µL) of culture medium were mixed with 540 µL of 0.1 M potassium phosphate buffer (pH 7.4) and 700 mg of glass beads YGB01 (0.1 mm, Yasui Kikai Corporation, Osaka, Japan). The cells in the mixture were disrupted using Multi-beads Shocker MB901U(S) (Yasui Kikai Corporation) at 4 °C with six cycles of 60 s each at 2500 rpm and intervals of 60 s. The mixture was then centrifuged at 21,600 × *g* for 5 min at 4 °C, and 10 µL of the supernatant was mixed with 5 µL of the loading buffer. This was then incubated at 95 °C for 15 min and a 5 µL sample was used for electrophoresis (200 V, 30 min).

### Analytical method

Quantification of 4-ABA and 4,3-AHBA in culture supernatants was performed using an HPLC system (Chromaster; Hitachi High-Tech Science Corporation, Tokyo, Japan) equipped with a reversed-phase column (L-column® ODS, 5 μm [4.6 × 150 mm]; Chemicals Evaluation and Research Institute, Tokyo, Japan) and a photodiode array detector (detection wavelength: 280 nm). Gradient elution (eluent A: 0.1 M KH_2_PO_4_ in 0.1% (v/v) H_3_PO_4_/H_2_O, eluent B: 70% (v/v) methanol/H_2_O) was performed at a flow rate of 1.0 mL/min and a column temperature of 40 °C. Quantification of glucose in culture supernatants was performed using the HPLC system equipped with an organic acid analytical column (ICSep ION-300, 5 μm [7.8 × 300 mm]; Tokyo Chemical Industry) and a refractive index detector. Elution (eluent: 5 mM H_2_SO_4_/H_2_O) was performed under isocratic conditions at a flow rate of 0.5 mL/min and a column temperature of 50 °C. The culture medium was diluted with 37 mM H_2_SO_4_ aqueous solution, and insoluble material was removed using AcroPrep™ Advance 96-well Filter Plates (350 µL, 0.2 μm WWPTFE; Nihon Pall Ltd., Tokyo, Japan). The resulting solution was allowed to stand to hydrolyze the *N*-glucosyl byproduct [17] before analysis. The percentage of 4,3-AHBA was calculated relative to the total concentration of 4,3-AHBA and 4-ABA.

### Colorimetric detection of 4,3-AHBA

The enzyme solution was prepared by mixing 0.1 g of laccase M120 powder (Amano Enzyme, Aichi, Japan) with 20 mL of 0.1 M sodium citrate buffer (pH 4.5), followed by centrifugation. The substrate solution was prepared by dissolving 16 mg of 4,3-AHBA in 20 mL of 0.1 M sodium citrate buffer (pH 4.5). In a 96-well assay plate, 5 µL of substrate solution was mixed with 195 µL of enzyme solution and allowed to stand for 20 min at room temperature. The absorbance was measured using Infinite® 200 PRO (TECAN, Männedorf, Switzerland). Thereafter, 100 µL of the resulting solution was mixed with 200 µL of methanol and filtered using AcroPrep™ Advance 96-well Filter Plates (350 µL, 0.2 μm WWPTFE). Molecular mass was analyzed using an LC-MS system (LCMS-2020; Shimadzu Co., Kyoto, Japan) equipped with a reversed-phase column (Sunrise C28 [2.0 × 150 mm]; ChromaNik Technologies, Osaka, Japan). Gradient elution (eluent A: 10 mM ammonium acetate aqueous solution, eluent B: 10 mM ammonium acetate in 99% (v/v) methanol/H_2_O) was performed at a flow rate of 0.2 mL/min and a column temperature of 40 °C. Electrospray ionization was performed in positive ion mode. Detection of 4,3-AHBA in the culture supernatant of *C. glutamicum* was performed by mixing 5 µL of the culture supernatant with 195 µL of the enzyme solution and measuring the absorbance at 446 nm.

### Random mutagenesis screening

KC551 was transformed with a solution of plasmids harboring randomly mutated genes, and each resulting single colony was individually cultured in 750 µL CGTG15 medium in deep-well plates at 30 °C with shaking at 800 rpm for 96 h. The colony that showed the highest absorbance at 446 nm, when the culture supernatant was mixed with the laccase solution, was selected as the strain containing the beneficial *Cv*PHBH mutant. The DNA fragment containing the gene region encoding the *Cv*PHBH mutant was amplified using PCR of DNA extracted from the strain as a template, and its sequence was confirmed.

### Structure modeling

A three-dimensional structural model was constructed using the SWISS-MODEL server [65] based on the crystal structure of *Pa*PHBH (PDB ID: 1IUT) bound to 4-ABA and FAD [16].

### Metabolic modeling

Model construction and FBA were performed using the COBRA Toolbox [66] and MATLAB R2023a (The MathWorks Inc., Massachusetts, USA) with the GNU Linear Programming Kit as a linear programming solver.

### Electronic supplementary material

Below is the link to the electronic supplementary material.

**Additional file 1: Table S1** Identity (%) matrix for amino acid sequences of PHBHs. **Table S2** Plasmids used in this study. **Table S3** Primers used in this study.

**Additional file 2: Fig. S1** Chromatograms for the culture supernatants of **A** KN001, **B** KN003, and **C** KN007 analyzed using HPLC. **Fig. S2** SDS-PAGE analysis of the cell lysate of KN001–007. **Fig. S3** Amino acid sequence alignment of *Cv*PHBH and *Pa*PHBH. **Fig. S4** Mass profile of the eluate with the absorption peak at ca. 446 nm in LC. **Fig. S5** Toxicity assay of 4,3-AHBA against *Corynebacterium glutamicum* NBRC 12168. **Fig. S6** Time variation of fermentation process parameters in fed-batch culture of *Corynebacterium glutamicum* strain KN034.

**Additional file 3**: Nucleotide sequences of the *pobA* genes codon-optimized for *Corynebacterium glutamicum*.

## Data Availability

All data generated or analyzed during this study are included in this published article and its supplementary information files.

## Abbreviations

4-ABA: 4-Aminobenzoic acid
ADC: 4-Amino-4-deoxychorismate
3,4-AHBA: 3-Amino-4-hydroxybenzoic acid
4,3-AHBA: 4-Amino-3-hydroxybenzoic acid
4-APA: 4-Aminophenylacetic acid
2,3,7-APOC: 2-Aminophenoxazin-3-one-7-carboxylic acid
DAHP: 3-Deoxy-D-arabino-heptulosonate-7-phosphate
FBA: Flux balance analysis
FPMO: Flavoprotein monooxygenase
4-HBA: 4-Hydroxybenzoic acid
4-HPA: 4-Hydroxyphenylacetic acid
HPAH: 4-Hydroxyphenylacetate 3-hydroxylase
PBO: Polybenzoxazole
PHBH: 4-Hydroxybenzoate 3-hydroxylase
