# Supplementary material for: A 4-hydroxybenzoate 3-hydroxylase mutant enables 4-amino-3-hydroxybenzoic acid production from glucose in Corynebacterium glutamicum
Source: Microb Cell Fact. 2023 Aug 29;22:168. doi: 10.1186/s12934-023-02179-y (PMC10466732; doi:10.1186/s12934-023-02179-y)
Supplement: Supplementary file 1 — Additional file 1: Table S1 Identity (%) matrix for amino acid sequences of PHBHs. Table S2 Plasmids used in this study. Table S3 Primers used in this study. [file 12934_2023_2179_MOESM1_ESM.pdf]

Additional file 1 (Supplementary Tables)

A 4-hydroxybenzoate 3-hydroxylase mutant enables 4-amino-3-hydroxybenzoic acid production from glucose in *Corynebacterium glutamicum*

Kyoshiro Nonaka[1*], Tatsuya Osamura[1], Fumikazu Takahashi[1]

[1]Biological Science Research, Kao Corporation, 1334 Minato, Wakayama, Wakayama 640-8580, Japan

[*]**Correspondence**: nonaka.kyoshiro@kao.com

Table S1 Identity (%) matrix for amino acid sequences of PHBHs. Analysis was performed using CLC Genomics Workbench v21.0.5 (Qiagen, Aarhus, Denmark) with default parameters

	***Rp*PHBH**	***Sm*PHBH**	***Cv*PHBH**	***Bd*PHBH**	***Cm*PHBH**	***Rf*PHBH**
***Rp*PHBH**		66	61	54	52	50
***Sm*PHBH**	66		62	51	50	47
***Cv*PHBH**	61	62		51	50	47
***Bd*PHBH**	54	51	51		62	51
***Cm*PHBH**	52	50	50	62		52
***Rf*PHBH**	50	47	47	51	52	

Table S2 Plasmids used in this study

Plasmid	Relevant characteristics
pHSG299	Km^R; *E. coli* cloning vector
pHM1519	Cryptic plasmid in *C. glutamicum* NBRC 12169
pKCG	Km^R; shuttle vector derived from pHSG299 and pHM1519
pKCG_P_{tuf}_T1	Km^R; pKCG harboring the expression cassette comprised of the constitutive P_{tuf} promoter and *rrnB* T1 terminator
pKCG_P_{tuf}_*Bd*PHBH_T1	Km^R; pKCG_P_{tuf}_T1 harboring a gene encoding PHBH from *B. diazoefficiens*
pKCG_P_{tuf}_*Cv*PHBH_T1	Km^R; pKCG_P_{tuf}_T1 harboring a gene encoding PHBH from *C. vibrioides*
pKCG_P_{tuf}_*Rp*PHBH_T1	Km^R; pKCG_P_{tuf}_T1 harboring a gene encoding PHBH from *R. palustris*
pKCG_P_{tuf}_*Sm*PHBH_T1	Km^R; pKCG_P_{tuf}_T1 harboring a gene encoding PHBH from *S. meliloti*
pKCG_P_{tuf}_*Cm*PHBH_T1	Km^R; pKCG_P_{tuf}_T1 harboring a gene encoding PHBH from *C. metallidurans*
pKCG_P_{tuf}_*Rf*PHBH_T1	Km^R; pKCG_P_{tuf}_T1 harboring a gene encoding PHBH from *R. fascians*
pKCG_P_{tuf}_*Cv*$PHBH^{Y201F}$_T1	Km^R; pKCG_P_{tuf}_T1 harboring a gene encoding Y201F mutant of *Cv*PHBH
pKCG_P_{tuf}_*Cv*$PHBH^{Y201S}$_T1	Km^R; pKCG_P_{tuf}_T1 harboring a gene encoding Y201S mutant of *Cv*PHBH
pKCG_P_{tuf}_*Cv*$PHBH^{Y201T}$_T1	Km^R; pKCG_P_{tuf}_T1 harboring a gene encoding Y201T mutant of *Cv*PHBH
pKCG_P_{tuf}_*Cv*$PHBH^{T294G}$_T1	Km^R; pKCG_P_{tuf}_T1 harboring a gene encoding T294G mutant of *Cv*PHBH
pKCG_P_{tuf}_*Cv*$PHBH^{T294A}$_T1	Km^R; pKCG_P_{tuf}_T1 harboring a gene encoding T294A mutant of *Cv*PHBH
pKCG_P_{tuf}_*Cv*$PHBH^{T294V}$_T1	Km^R; pKCG_P_{tuf}_T1 harboring a gene encoding T294V mutant of *Cv*PHBH
pKCG_P_{tuf}_*Cv*$PHBH^{T294L}$_T1	Km^R; pKCG_P_{tuf}_T1 harboring a gene encoding T294L mutant of *Cv*PHBH
pKCG_P_{tuf}_*Cv*$PHBH^{T294I}$_T1	Km^R; pKCG_P_{tuf}_T1 harboring a gene encoding T294I mutant of *Cv*PHBH
pKCG_P_{tuf}_*Cv*$PHBH^{T294S}$_T1	Km^R; pKCG_P_{tuf}_T1 harboring a gene encoding T294S mutant of *Cv*PHBH
pKCG_P_{tuf}_*Cv*$PHBH^{T294C}$_T1	Km^R; pKCG_P_{tuf}_T1 harboring a gene encoding T294C mutant of *Cv*PHBH
pKCG_P_{tuf}_*Cv*$PHBH^{Y201F/T294S}$_T1	Km^R; pKCG_P_{tuf}_T1 harboring a gene encoding Y201F/T294S mutant of *Cv*PHBH
pKCG_P_{tuf}_*Cv*$PHBH^{Y161S/D357V}$_T1	Km^R; pKCG_P_{tuf}_T1 harboring a gene encoding Y161S/D357V mutant of *Cv*PHBH
pKCG_P_{tuf}_*Cv*$PHBH^{Y161S}$_T1	Km^R; pKCG_P_{tuf}_T1 harboring a gene encoding Y161S mutant of *Cv*PHBH
pKCG_P_{tuf}_*Cv*$PHBH^{D357V}$_T1	Km^R; pKCG_P_{tuf}_T1 harboring a gene encoding D357V mutant of *Cv*PHBH
pKCG_P_{tuf}_*Cv*$PHBH^{M106G}$_T1	Km^R; pKCG_P_{tuf}_T1 harboring a gene encoding M106G mutant of *Cv*PHBH
pKCG_P_{tuf}_*Cv*$PHBH^{M106A}$_T1	Km^R; pKCG_P_{tuf}_T1 harboring a gene encoding M106A mutant of *Cv*PHBH
pKCG_P_{tuf}_*Cv*$PHBH^{M106V}$_T1	Km^R; pKCG_P_{tuf}_T1 harboring a gene encoding M106V mutant of *Cv*PHBH
pKCG_P_{tuf}_*Cv*$PHBH^{M106L}$_T1	Km^R; pKCG_P_{tuf}_T1 harboring a gene encoding M106L mutant of *Cv*PHBH
pKCG_P_{tuf}_*Cv*$PHBH^{M106I}$_T1	Km^R; pKCG_P_{tuf}_T1 harboring a gene encoding M106I mutant of *Cv*PHBH
pKCG_P_{tuf}_*Cv*$PHBH^{M106S}$_T1	Km^R; pKCG_P_{tuf}_T1 harboring a gene encoding M106S mutant of *Cv*PHBH
pKCG_P_{tuf}_*Cv*$PHBH^{M106T}$_T1	Km^R; pKCG_P_{tuf}_T1 harboring a gene encoding M106T mutant of *Cv*PHBH

pKCG_P_{tuf}_*Cv*PHBH^{M106C}_T1	Km^R; pKCG_P_{tuf}_T1 harboring a gene encoding M106C mutant of *Cv*PHBH
pKCG_P_{tuf}_*Cv*PHBH$^{M106A/T294S}$_T1	Km^R; pKCG_P_{tuf}_T1 harboring a gene encoding M106A/T294S mutant of *Cv*PHBH
pKCG_P_{tuf}_*Cv*PHBH$^{M106A/Y201F/T294S}$_T1	Km^R; pKCG_P_{tuf}_T1 harboring a gene encoding M106A/Y201F/T294S mutant of *Cv*PHBH
pHKP*sacB*1	Km^R; suicide vector for two-step homologous recombination containing the *sacB* gene from *B. subtilis*
pHKP*sacB*1_P_{tuf}_*aroG*	Km^R; pHKP*sacB*1 harboring P_{tuf} inserted inside the homologous region to the *aroG* (cg2391) locus
pHKP*sacB*1_P_{tuf}_*aroE3*	Km^R; pHKP*sacB*1 harboring P_{tuf} inserted inside the homologous region to the *aroE3* (cg1835) locus
pHKP*sacB*1_P_{tuf}_*aroB*	Km^R; pHKP*sacB*1 harboring P_{tuf} inserted inside the homologous region to the *aroB* (cg1827) locus
pHKP*sacB*1_P_{tuf}_*aroA*	Km^R; pHKP*sacB*1 harboring P_{tuf} inserted inside the homologous region to the *aroA* (cg0873) locus
pHKP*sacB*1_Δ*pobA*_P_{tuf}_*qsuC*	Km^R; pHKP*sacB*1 harboring P_{tuf} and the *qsuC* (cg0503) gene inserted inside the homologous region to the *pobA* (cg1226) locus
pHKP*sacB*1_P_{tuf}_*aroC*	Km^R; pHKP*sacB*1 harboring P_{tuf} inserted inside the homologous region to the *aroC* (cg1829) locus
pHKP*sacB*1_P_{tuf}_*tkt*	Km^R; pHKP*sacB*1 harboring P_{tuf} inserted inside the homologous region to the *tkt* (cg1774) locus
pHKP*sacB*1_P_{tuf}_*ppsA*	Km^R; pHKP*sacB*1 harboring P_{tuf} inserted inside the homologous region to the *ppsA* (cg0644) locus
pHKP*sacB*1_Δ*qsuB*	Km^R; pHKP*sacB*1 harboring the homologous region to the *qsuB* (cg0502) locus
pHKP*sacB*1_P_{tuf}_*pabAB*	Km^R; pHKP*sacB*1 harboring P_{tuf} inserted inside the homologous region to the *pabAB* (cg1134) locus
pKCG_P_{tuf}_*aroG*^{D146N}_T1	Km^R; pKCG_P_{tuf}_T1 harboring *aroG*^{D146N}
pHKP*sacB*1_P_{tuf}_*aroG*^{D146N}	Km^R; pHKP*sacB*1 harboring the expression cassette P_{tuf}_*aroG*^{D146N}_T1 inserted inside the homologous region to the cg2567 locus
pKCG_P_{tuf}_*aroF*_T1	Km^R; pKCG_P_{tuf}_T1 harboring *aroF* (cg1129)
pKCG_P_{tuf}_*aroF*^{P155L}_T1	Km^R; pKCG_P_{tuf}_T1 harboring *aroF*^{P155L}
pHKP*sacB*1_P_{tuf}_*aroF*^{P155L}	Km^R; pHKP*sacB*1 harboring the expression cassette P_{tuf}_*aroF*^{P155L}_T1 inserted inside the homologous region to the cg2088 locus

Table S3 Primers used in this study

Primer name	Sequence (5′ to 3′)
pHSG299-F	AAGGACTGCTCCCAATACGGTTATCCACAGAATCA
pHSG299-R	GGGCGATCAGCGACGACTGGCCGTCGTTTTACAAC
pHM1519-F	CGTCGCTGATCGCCCTCGCGAC
pHM1519-R	TTGGGAGCAGTCCTTGTGCGCTTACGAG
pKCG-vec-F	CGTCGCTGATCGCCCTCGCGAC
pKCG-vec-R	GGATCTAAACGATCTACTGGCCGTCGTTTTACAAC
Ptuf-F	AGATCGTTTAGATCCGAAGGAAAACGTCGAAAAGC
Ptuf-R	TGTATGTCCTCCTGGACTTCGTGGTGGCTAC
T1-F	CCAGGAGGACATACAGGTAGTGTGGGGTCTCCCCA
T1-R	GGGCGATCAGCGACGAAATCCGCTCCCGGCGGATT
pKCG_Ptuf_T1-vec-F	GGTAGTGTGGGGTCTCCCCATGC
pKCG_Ptuf_T1-vec-R	TGTATGTCCTCCTGGACTTCGTGGTGGCTAC
BdPHBH-F	CCAGGAGGACATACAATGCGTACTCAGGTGGGAAT
BdPHBH-R	AGACCCCACACTACCTTAAGCAAGTGGCATGCCTA
CvPHBH-F	CCAGGAGGACATACAATGCGCACTCAGGTGGCTAT
CvPHBH-R	AGACCCCACACTACCTTATACGAGTGGCAGTCCTA
RpPHBH-F	CCAGGAGGACATACAATGCGCACTCAGGTGGCAAT
RpPHBH-R	AGACCCCACACTACCTTAGTATGGCAGGCCTACGT
SmPHBH-F	CCAGGAGGACATACAATGCGCACCCAAGTGGTCAT
SmPHBH-R	AGACCCCACACTACCTTAGAACGGCAGACCCACGT
CmPHBH-F	CCAGGAGGACATACAATGCGCACTCAGGTTGGTAT
CmPHBH-R	AGACCCCACACTACCTTAGTGGCTCAGTCCAACCA
RfPHBH-F	CCAGGAGGACATACAATGCGTACCCAAGTGGCCAT
RfPHBH-R	AGACCCCACACTACCTTAGAAGCCAATCGGAAGGC
CvPHBH-Y201F-F	TTGATC**TTC**TCGAACCATGATCGCGGT
CvPHBH-Y201F-R	GTTCGA**GAA**GATCAACTCGTGGTCACA
CvPHBH-Y201S-F	TTGATC**TCT**TCGAACCATGATCGCGGT
CvPHBH-Y201S-R	GTTCGA**AGA**GATCAACTCGTGGTCACA
CvPHBH-Y201T-F	TTGATC**ACA**TCGAACCATGATCGCGGT
CvPHBH-Y201T-R	GTTCGA**TGT**GATCAACTCGTGGTCACA
CvPHBH-T294G-F	CCACCC**GGT**GGAGCGAAAGGGATGAAC
CvPHBH-T294G-R	CGCTCC**ACC**GGGTGGAACGATATGAGC
CvPHBH-T294A-F	CCACCC**GCA**GGAGCGAAAGGGATGAAC
CvPHBH-T294A-R	CGCTCC**TGC**GGGTGGAACGATATGAGC
CvPHBH-T294V-F	CCACCC**GTT**GGAGCGAAAGGGATGAAC

CvPHBH-T294V-R	CGCTCC**AAC**GGGTGGAACGATATGAGC
CvPHBH-T294L-F	CCACCC**CTG**GGAGCGAAAGGGATGAAC
CvPHBH-T294L-R	CGCTCC**CAG**GGGTGGAACGATATGAGC
CvPHBH-T294I-F	CCACCC**ATT**GGAGCGAAAGGGATGAAC
CvPHBH-T294I-R	CGCTCC**AAT**GGGTGGAACGATATGAGC
CvPHBH-T294S-F	CCACCC**TCT**GGAGCGAAAGGGATGAAC
CvPHBH-T294S-R	CGCTCC**AGA**GGGTGGAACGATATGAGC
CvPHBH-T294C-F	CCACCC**TGT**GGAGCGAAAGGGATGAAC
CvPHBH-T294C-R	CGCTCC**ACA**GGGTGGAACGATATGAGC
CvPHBH-Y161S-F	GACGGC**TCC**CACGGAGTTTCTCGTGCG
CvPHBH-Y161S-R	TCCGTG**GGA**GCCGTCGCAGCCGGCAAT
CvPHBH-D357V-F	GACCAG**GTC**GGCTTCGACCGCAAGATG
CvPHBH-D357V-R	GAAGCC**GAC**CTGGTCTGGGAAGCGGTG
CvPHBH-M106G-F	GAGGTG**GGT**AAGGACCTGTTTGATGCA
CvPHBH-M106G-R	GTCCTT**ACC**CACCTCCTGTTGGCCGTA
CvPHBH-M106A-F	GAGGTG**GCA**AAGGACCTGTTTGATGCA
CvPHBH-M106A-R	GTCCTT**TGC**CACCTCCTGTTGGCCGTA
CvPHBH-M106V-F	GAGGTG**GTT**AAGGACCTGTTTGATGCA
CvPHBH-M106V-R	GTCCTT**AAC**CACCTCCTGTTGGCCGTA
CvPHBH-M106L-F	GAGGTG**CTG**AAGGACCTGTTTGATGCA
CvPHBH-M106L-R	GTCCTT**CAG**CACCTCCTGTTGGCCGTA
CvPHBH-M106I-F	GAGGTG**ATT**AAGGACCTGTTTGATGCA
CvPHBH-M106I-R	GTCCTT**AAT**CACCTCCTGTTGGCCGTA
CvPHBH-M106S-F	GAGGTG**TCT**AAGGACCTGTTTGATGCA
CvPHBH-M106S-R	GTCCTT**AGA**CACCTCCTGTTGGCCGTA
CvPHBH-M106T-F	GAGGTG**ACA**AAGGACCTGTTTGATGCA
CvPHBH-M106T-R	GTCCTT**TGT**CACCTCCTGTTGGCCGTA
CvPHBH-M106C-F	GAGGTG**TGT**AAGGACCTGTTTGATGCA
CvPHBH-M106C-R	GTCCTT**ACA**CACCTCCTGTTGGCCGTA
epPCR-ins-F	CACGAAGTCCAGGAGGACATACAATG
epPCR-ins-R	CATGGGGAGACCCCACACTACCTTA
epPCR-vec-F	TGGGGTCTCCCCATGCGAGAGTAG
epPCR-vec-R	CTCCTGGACTTCGTGGTGGCTAC
epPCR-amp-F	CGACGTTGTAAAACGACGGCCAGT
epPCR-amp-R	GGGCGATCAGCGACGAAATCCG
pHKPsacB1-vec-F	TCGACTCTAGAGGATCTACT
pHKPsacB1-vec-R	ACTGGCCGTCGTTTTACAAC
aroG(cg2391)-up-F	AAAACGACGGCCAGTCCAACTTGGTAGTCCTGGGT

aroG(cg2391)-up-R	GGATCTAAACGATCTACCCCTATTCATAGCACGAT
aroG(cg2391)-dw-F	CCAGGAGGACATACAGTGAGTTGGACAGTTGATAT
aroG(cg2391)-dw-R	ATCCTCTAGAGTCGATGGTCAAACGGCCAGGCTCG
aroE3(cg1835)-up-F	AAAACGACGGCCAGTATCCCTGAAGGTTCCTCCAT
aroE3(cg1835)-up-R	GGATCTAAACGATCTTTTTATTCGCTGATCCTTAT
aroE3(cg1835)-dw-F	CCAGGAGGACATACAATGGGTTCTCACATCACTCA
aroE3(cg1835)-dw-R	ATCCTCTAGAGTCGACCTCCTTGACCAAGGACGTG
aroB(cg1827)-up-F	AAAACGACGGCCAGTGTTCCTGGATGACAACGGCG
aroB(cg1827)-up-R	GGATCTAAACGATCTGGGGCACGTTGCCTTTCGCT
aroB(cg1827)-dw-F	CCAGGAGGACATACAATGAGCGCAGTGCAGATTTT
aroB(cg1827)-dw-R	ATCCTCTAGAGTCGATGTTGCCGTCGCGGTTTTTC
aroA(cg0873)-up-F	AAAACGACGGCCAGTCACCACCGCCTACGCTCCAA
aroA(cg0873)-up-R	GGATCTAAACGATCTAATAGATGTTATGTGTGGAA
aroA(cg0873)-dw-F	CCAGGAGGACATACAATGGTCTTTGTGTCTGATTC
aroA(cg0873)-dw-R	ATCCTCTAGAGTCGACTGTCGACGCCAACGCAGCC
pobA(cg1226)-up-F	AAAACGACGGCCAGTGTGGTCATTTATACCTCGCG
pobA(cg1226)-up-R	GGATCTAAACGATCTGGGGAACTCCTTTCATTGAC
pobA(cg1226)-dw-F	AATCTCAAAAAGTAGACGCTCGTGTCAACGACCAC
pobA(cg1226)-dw-R	ATCCTCTAGAGTCGAGGACAGCAAGCAACGCGAGG
qsuC(cg0503)-orf-F	CCAGGAGGACATACAATGCCTGGAAAAATTCTCCT
qsuC(cg0503)-orf-R	CTACTTTTTGAGATTTGCCA
aroC(cg1829)-up-F	AAAACGACGGCCAGTACAAACATGGAGGCAATTAG
aroC(cg1829)-up-R	GGATCTAAACGATCTTATCGCACGAAAGGCCGCAG
aroC(cg1829)-dw-F	CCAGGAGGACATACAATGCTAGGCATGCTTCGATG
aroC(cg1829)-dw-R	ATCCTCTAGAGTCGATTTTCAGGGCGCGAGGCACA
tkt(cg1774)-up-F	AAAACGACGGCCAGTACATTTTCTAAGACACACGG
tkt(cg1774)-up-R	GGATCTAAACGATCTTCCTTCCTGGGTTAAACCGG
tkt(cg1774)-dw-F	CCAGGAGGACATACAATGACCACCTTGACGCTGTC
tkt(cg1774)-dw-R	ATCCTCTAGAGTCGACGTAAGCCTTGTAACGAGCA
ppsA(cg0644)-up-F	AAAACGACGGCCAGTATACTCCGGATTATCAGCTC
ppsA(cg0644)-up-R	GGATCTAAACGATCTGGTGTCTCCTTATTTAATAA
ppsA(cg0644)-dw-F	CCAGGAGGACATACAATGACCAACAGTTTGAACAT
ppsA(cg0644)-dw-R	ATCCTCTAGAGTCGACGTTGGAGTGGATAGTTTCC
qsuB(cg0502)-up-F	AAAACGACGGCCAGTGGGACTGTCTGCATCCGGTG
qsuB(cg0502)-up-R	GGCTAGTTCGGGATGTTACTGAGCCGCGAGTTGGG
qsuB(cg0502)-dw-F	CATCCCGAACTAGCCCCCCA
qsuB(cg0502)-dw-R	ATCCTCTAGAGTCGATGCCAGTGCCTGCGCGCGGG
pabAB(cg1134)-up-F	AAAACGACGGCCAGTTGTCTTTGGCTCATGGTGGT

pabAB(cg1134)-up-R	GGATCTAAACGATCTGCACCGCCAGGCCGAAGCCG
pabAB(cg1134)-dw-F	CCAGGAGGACATACAATGCGCGTTTTAATTATTGA
pabAB(cg1134)-dw-R	ATCCTCTAGAGTCGAAAATTGCACACCCCACTGCG
aroGec-F	CCAGGAGGACATACAATGAACTACCAGAACGACGA
aroGec-R	AGACCCCACACTACCTTAACCGCGACGGGCTTTGA
Ptuf-T1-cassette-F	AGATCGTTTAGATCCGAAGG
Ptuf-T1-cassette-R	AAATCCGCTCCCGGCGGATTTGTCCTACTCAGGAG
cg2567-up-F	AAAACGACGGCCAGTAACAACACCTACAAGCTGAC
cg2567-up-R	GGATCTAAACGATCTGGGGTTTAGCTTTCAGTGCA
cg2567-dw-F	GCCGGGAGCGGATTTAAATCCTGTGCCGTGAGGTT
cg2567-dw-R	ATCCTCTAGAGTCGACGTGGCTTCAGCACCTGCGG
aroF-F	CCAGGAGGACATACAATGAGTTCTCCAGTCTCACT
aroF-R	AGACCCCACACTACCTTACTTGGCTGCTGCTCGGC
aroF-P155L-F	CTCGA**GCT**AAACAGCCCTCA
aroF-P155L-R	GTTT**AGC**TCGAGGAATTCGC
cg2088-up-F	AAAACGACGGCCAGTTCAGTGAGTCGCTGGTTGCT
cg2088-up-R	GGATCTAAACGATCTGAAGGCCAGACCATTTCTCT
cg2088-dw-F	GCCGGGAGCGGATTTTCAGAACTGCTCAACAAAGA
cg2088-dw-R	ATCCTCTAGAGTCGAGGGCTTTTCTGGATCAAGAT

Underlined text: overlapping sequences for In-Fusion cloning.

Bold text: sequences modified to introduce mutations.
