# Supplementary material for: A 4-hydroxybenzoate 3-hydroxylase mutant enables 4-amino-3-hydroxybenzoic acid production from glucose in Corynebacterium glutamicum
Source: Microb Cell Fact. 2023 Aug 29;22:168. doi: 10.1186/s12934-023-02179-y (PMC10466732; doi:10.1186/s12934-023-02179-y)
Supplement: Supplementary file 2 — Additional file 2: Fig. S1 Chromatograms for the culture supernatants of A KN001, B KN003, and C KN007 analyzed using HPLC. Fig. S2 SDS-PAGE analysis of the cell lysate of KN001–007. Fig. S3 Amino acid sequence alignment of CvPHBH and PaPHBH. Fig. S4 Mass profile of the eluate with the absorption peak at ca. 446 nm in LC. Fig. S5 Toxicity assay of 4,3-AHBA against Corynebacterium glutamicum NBRC 12168. Fig. S6 Time variation of fermentation process parameters in fed-batch culture of Corynebacterium glutamicum strain KN034. [file 12934_2023_2179_MOESM2_ESM.pdf]

Additional file 2 (Supplementary Figures)

A 4-hydroxybenzoate 3-hydroxylase mutant enables 4-amino-3-hydroxybenzoic acid production from glucose in *Corynebacterium glutamicum*

Kyoshiro Nonaka[1*], Tatsuya Osamura[1], Fumikazu Takahashi[1]

[1]Biological Science Research, Kao Corporation, 1334 Minato, Wakayama, Wakayama 640-8580, Japan

[*]**Correspondence**: nonaka.kyoshiro@kao.com

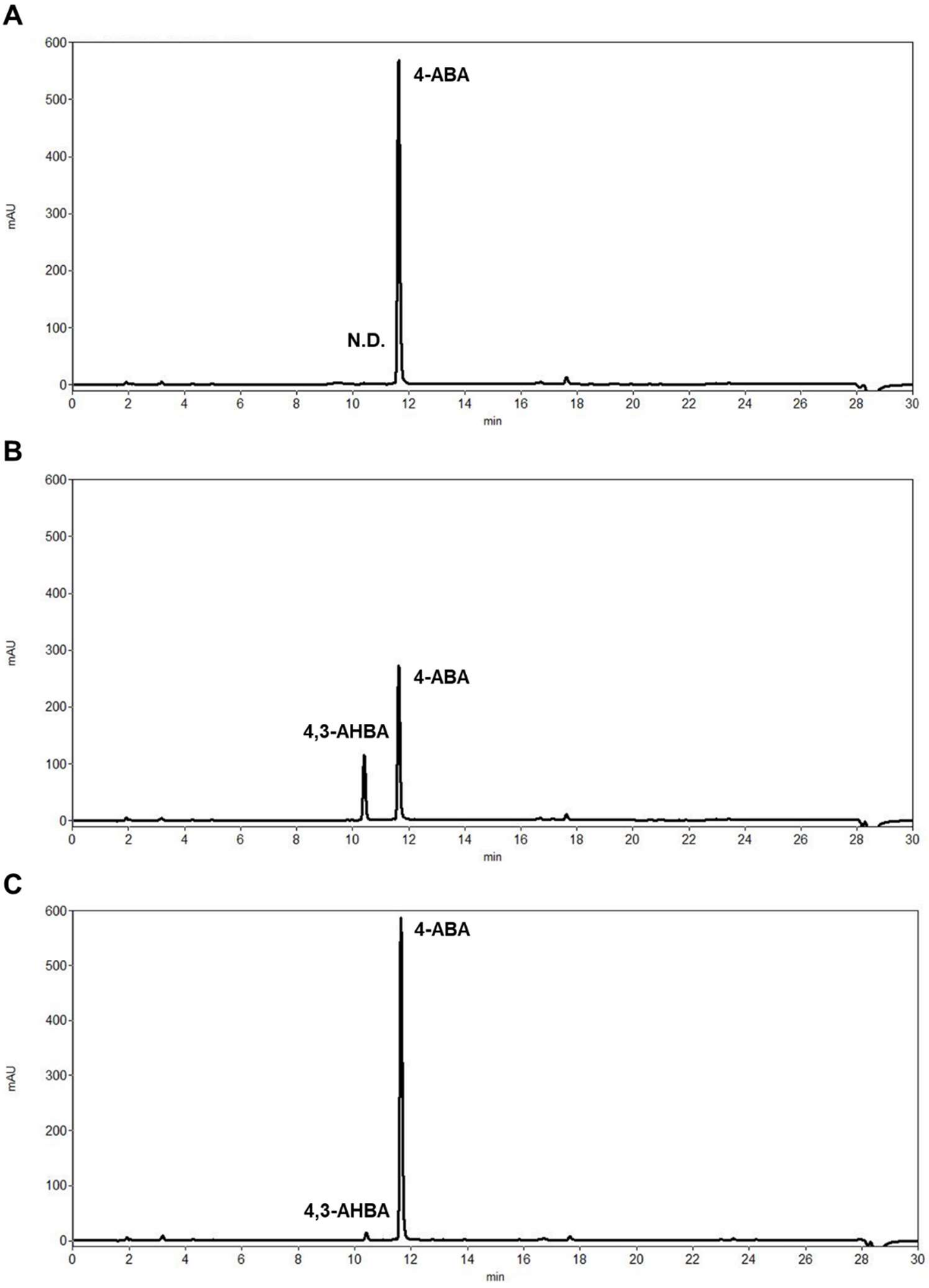

Fig. S1 Chromatograms for the culture supernatants of **A** KN001, **B** KN003, and **C** KN007 analyzed using HPLC. Detection was performed by measuring absorbance at 280 nm. The peak corresponding to 4,3-AHBA was not detected (N.D.) in KN001.

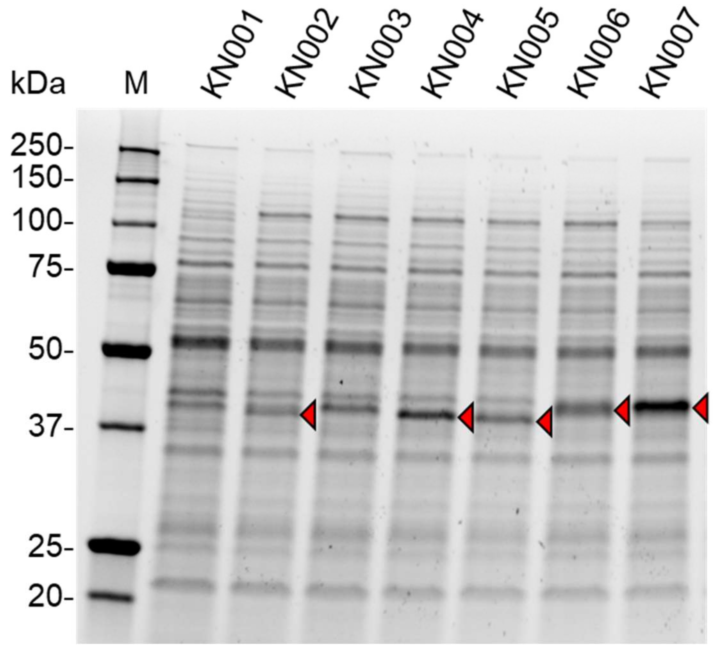

Fig. S2 SDS-PAGE analysis of the cell lysate of KN001–007. The supernatants after bead disruption of the cells were mixed with sample buffer without purification and loaded onto the gel. Red arrowheads indicate additional bands corresponding to heterologously expressed PHBHs.

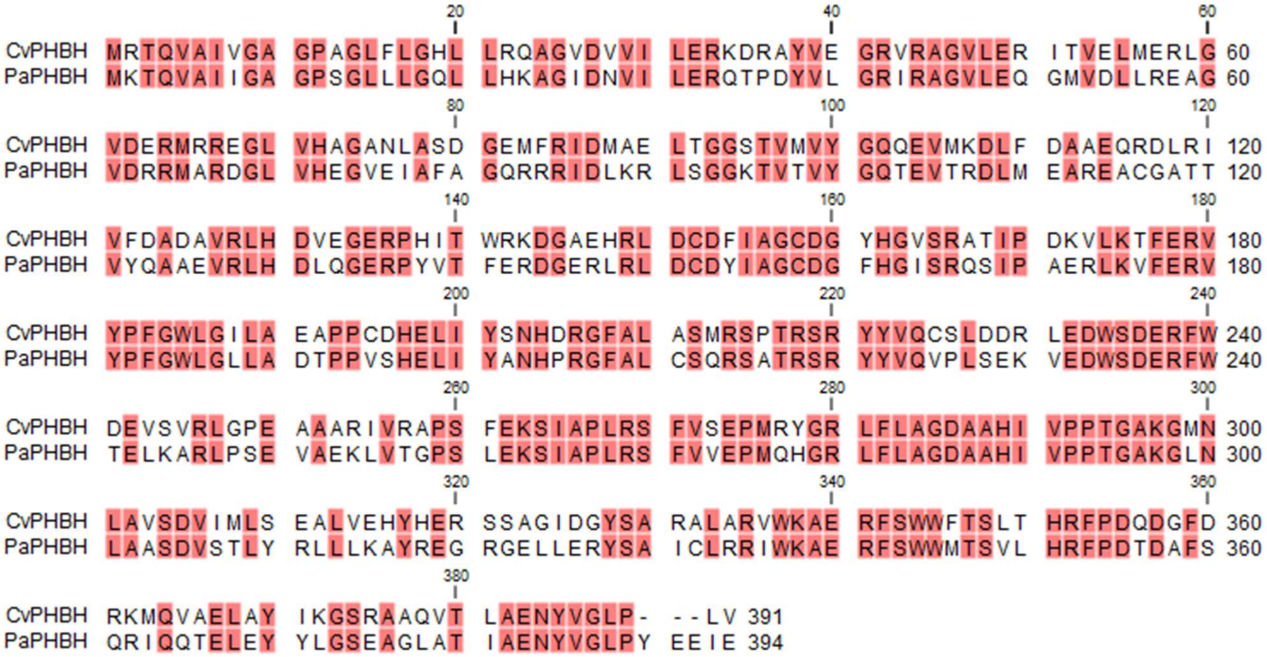

Fig. S3 Amino acid sequence alignment of *Cv*PHBH and *Pa*PHBH. The text in red represents the conserved residues.

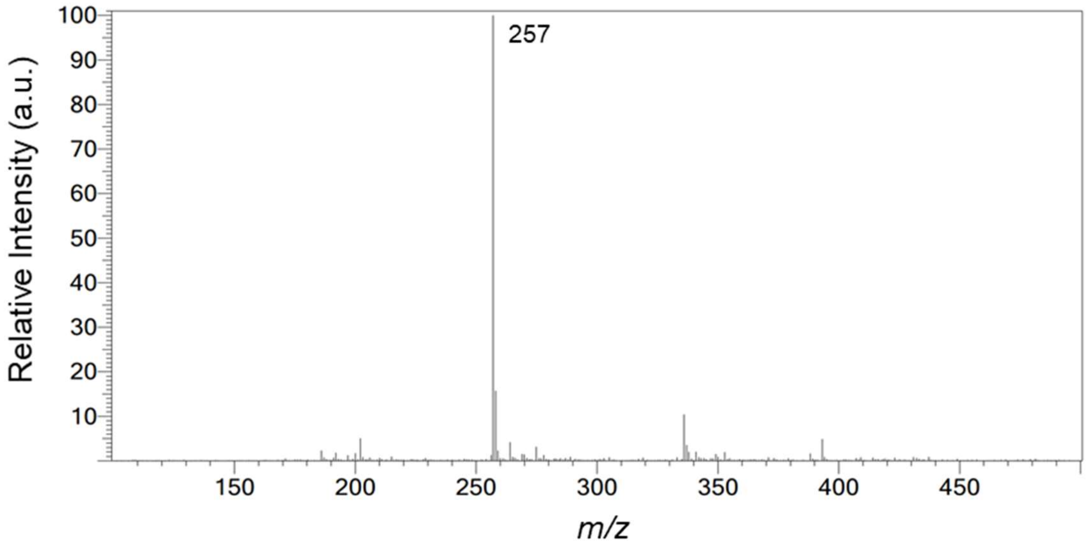

Fig. S4 Mass profile of the eluate with the absorption peak at ca. 446 nm in LC. The major molecular mass peak is *m/z* = 257, indicating the formation of 2,3,7-APOC ($[M + H]^+$ = 257).

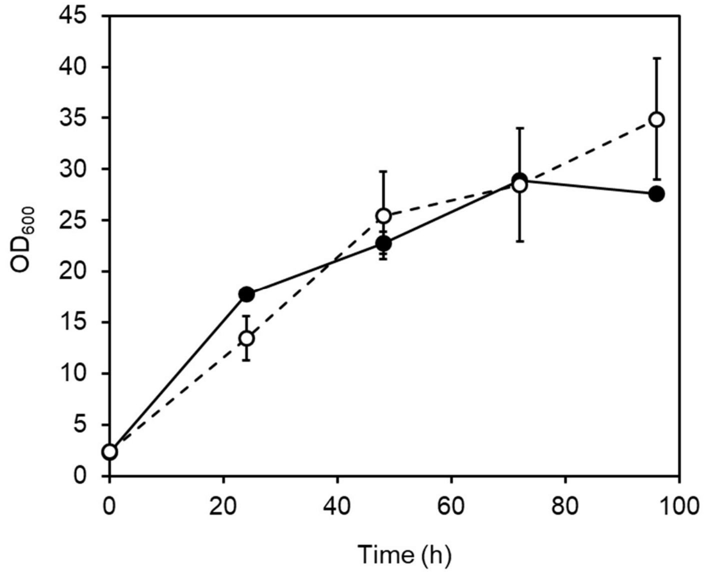

Fig. S5 Toxicity assay of 4,3-AHBA against *Corynebacterium glutamicum* NBRC 12168. Filled and open circles represent cultures containing 0 and 100 mM 4,3-AHBA, respectively. Data are represented as the mean ± standard deviation of biological replicates (n = 4). The strain was grown at 30 °C and 800 rpm in 750 µL of CGTG15 medium containing 4,3-AHBA at various concentrations (0, 100, 200, 300, and 400 mM), and cell growth was monitored by measuring OD_{600} values every 24 h. To correct for color changes in the cell culture medium, the OD_{600} value of the culture supernatant was subtracted from the OD_{600} value of the corresponding culture medium at each time point. In the case of cultures containing more than 200 mM 4,3-AHBA, OD_{600} values could not be measured owing to strong coloration and precipitation caused by spontaneous oxidation of 4,3-AHBA.

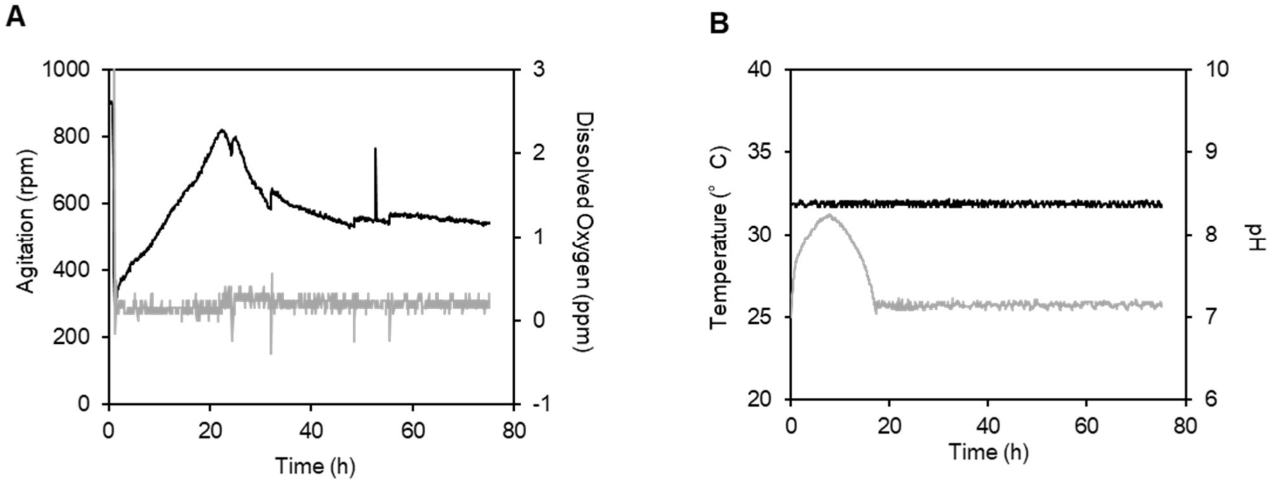

Fig. S6 Time variation of fermentation process parameters in fed-batch culture of *Corynebacterium glutamicum* strain KN034. **A** Agitation (black line) and dissolved oxygen (gray line). **B** Temperature (black line) and pH (gray line).
